# Small molecule-assisted synthesis of carbon supported platinum intermetallic fuel cell catalysts

**DOI:** 10.1038/s41467-022-34037-7

**Published:** 2022-10-31

**Authors:** Tian-Wei Song, Cong Xu, Zhu-Tao Sheng, Hui-Kun Yan, Lei Tong, Jun Liu, Wei-Jie Zeng, Lu-Jie Zuo, Peng Yin, Ming Zuo, Sheng-Qi Chu, Ping Chen, Hai-Wei Liang

**Affiliations:** 1 Hefei National Research Center for Physical Sciences at the Microscale, Department of Chemistry, University of Science and Technology of China, Hefei, 230026 China; 2 College of Chemistry and Materials Science, Anhui Normal University, Wuhu, 241000 China; 3 Institute of Solid State Physics, Hefei Institutes of Physical Science, Chinese Academy of Sciences, Hefei, 230031 China; 4 Anhui Contango New Energy Technology Co., Ltd, Hefei, 230088 China; 5 Institute of High Energy Physics, Chinese Academy of Sciences, Beijing, 100049 China; 6 School of Chemistry and Chemical Engineering, Anhui University, Hefei, 230601 China

**Keywords:** Fuel cells, Electrocatalysis, Fuel cells

## Abstract

Supported ordered intermetallic compounds exhibit superior catalytic performance over their disordered alloy counterparts in diverse reactions. But the synthesis of intermetallic compounds catalysts often requires high-temperature annealing that leads to the sintering of metals into larger crystallites. Herein, we report a small molecule-assisted impregnation approach to realize the general synthesis of a family of intermetallic catalysts, consisting of 18 binary platinum intermetallic compounds supported on carbon blacks. The molecular additives containing heteroatoms (that is, O, N, or S) can be coordinated with platinum in impregnation and thermally converted into heteroatom-doped graphene layers in high-temperature annealing, which significantly suppress alloy sintering and insure the formation of small-sized intermetallic catalysts. The prepared optimal PtCo intermetallics as cathodic oxygen-reduction catalysts exhibit a high mass activity of 1.08 A mg_Pt_^–1^ at 0.9 V in H_2_-O_2_ fuel cells and a rated power density of 1.17 W cm^–2^ in H_2_-air fuel cells.

## Abstract (web summary)

Synthesis of small sized Pt intermetallic catalysts remains challenging. Herewith authors prepared 18 binary Pt intermetallic compounds with small particle size by molecule-assisted synthesis strategy to in-situ form the heteroatom-doped carbon shell.

## Introduction

Intermetallic compounds (IMCs), as a special kind of alloy materials with heterometallic bonding, defined stoichiometry, and long-range atomically ordered crystal structure, have raised sustained research interests in diverse catalytic fields including chemical transformations, organic synthesis, gas purification, and energy conversion^1–3^. Particularly, carbon supported Pt-M (M is a base metal, typically a 3*d* transition metal) IMCs represent one of the most promising low-Pt electrocatalysts for the sluggish oxygen-reduction reaction (ORR)^4–6^, a bottleneck restricting the large-scale commercialization of proton-exchange-membrane fuel cells (PEMFCs) vehicles. For catalyzing ORR, Pt-IMCs catalysts are distinctly advantageous over their disordered counterparts in activity enhancement, which can be ascribed to the more pronounced ligand and/or strain effects in ordered IMCs structures^7–12^. Moreover, the strong 3*d*–5*d* orbital interactions between M and Pt lead to a larger formation enthalpy in IMCs relative to disordered alloys, resulting in enhanced structural stability of the IMCs catalysts under corrosive fuel cell conditions^13,14^.

In thermodynamics, the Gibbs’ free energy ($\triangle {G}_{d\to o}^{\infty }$) change in disorder-to-order phase-transition depends on the temperature (*T*), the change of enthalpy ($\triangle {H}_{d\to o}$), and the entropy ($\triangle {S}_{d\to o}$)^15^:

1 $$\triangle {G}_{d\to o}^{\infty }=\triangle {H}_{d\to o}-T\triangle {S}_{d\to o}$$

where *d* and *o* indicate the disordered and ordered structures, respectively. The stronger Pt-M heterometallic bonding energy in ordered structures relative to disordered ones makes $\triangle {H}_{d\to o}$ negative, while $\triangle {S}_{d\to o}$ is also negative in the atom ordering process. Accordingly, the ordered intermetallics are thermodynamically favorable to generate relative to disordered solid solutions below the phase-transition temperature. In kinetics, the realization of the disorder-to-order transition, however, must overcome the high energy barrier of atom diffusion and ordering in solid states. Although direct low-temperature solution syntheses and electrochemical methods are effective for preparing a few low-melting-point Pt and Pd-based intermetallics^9,16–18^, the synthesis of most Pt-IMCs catalysts generally requires a high-temperature thermal annealing process to promote atomic ordering, which inevitably leads to metal sintering and makes it challenging to produce nano-sized IMCs catalysts with high surface areas and mass-based activities for practical applications^5,19,20^.

Recent efforts aimed at preparing small Pt-IMCs nanoparticle catalysts, particularly for fuel cell application^5,21–26^, include coating disordered alloy nanoparticles with carbons or metal oxide protective shells prior to high-temperature annealing^27–31^, KCl matrix-assisted annealing^32^, heteroatom-doped carbons anchoring synthesis^6,33^, low-temperature chemical vapor deposition with organometallic precursors^34^, and thermal decomposition of heterometallic compound precursors^35^. Yet, despite these efforts, a more effective method needs to be developed to break the trade-off between small particle size (thus high electrochemically active surface areas) and high ordering degree of Pt intermetallic catalysts for the further PEMFCs performance improvement^36,37^.

Here, we develop a small molecule-assisted impregnation approach to realize the grams scale synthesis of small-sized and highly ordered Pt-IMCs catalysts with commercial carbon black supports. The molecular additives containing coordinating heteroatoms (that is, O, N, or S) can greatly suppress the metal particle sintering in high-temperature H_2_-annealing synthesis, by which the alloy nanoparticles evolve into intermetallic phases but do not suffer from severe sintering, guaranteeing the formation of small-sized IMCs nanoparticle catalysts with high ordering degree. With the optimal sodium thioglycolate additive, we demonstrate the combinatorial synthesis of catalyst libraries consisting of 18 binary Pt-IMCs. We understand that small molecule additives are coordinated with platinum in impregnation and thermally converted into heteroatom-doped carbon shells around alloy particles, which significantly suppress particle sintering in high-temperature annealing. The prepared PtCo intermetallic catalysts exhibit a high mass activity (MA) of 1.08 A mg_Pt_^–1^ at 0.9 V_iR-correct_ in H_2_–O_2_ fuel cells and a large rated power density of 1.17 W cm^–2^ at 94 °C in H_2_-air fuel cells, along with an outstanding durability (MA and rated power density remained 75% and 88%, respectively, after 30,000 cycles’ accelerated durability tests (ADT)).

## Results

### Screening molecule additives

**Fig. 1:**
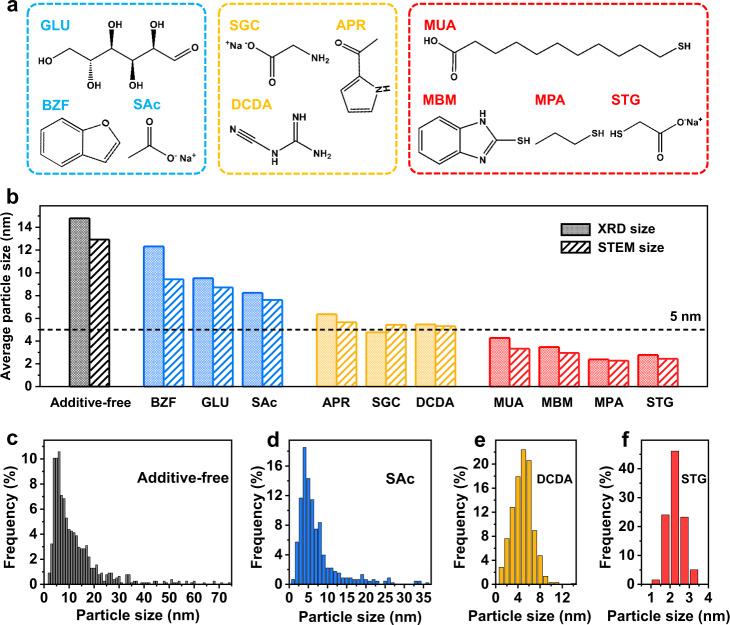
Screening small molecules for suppressing metal sintering. **a** The structures of the molecules used as additives for suppressing metal sintering. **b** XRD (solid shadow column) and STEM (twill stripes column) sizes of PtCo particles with various molecule additives used for the catalyst synthesis at 700 °C. STEM statistics of particle size distribution of PtCo synthesized without (**c**) and with three representative molecule additives, including SAc (**d**), DCDA (**e**), and STG (**f**).

We first screened a diversity of small molecule additives containing coordinating heteroatoms (that is, O, N, or S) for suppressing metal sintering in high-temperature H_2_-annealing (Fig. 1a), by taking PtCo as examples. Molecule additives and metal precursors (i.e. H_2_PtCl_6_ and CoCl_2_) were wet-impregnated onto carbon black supports (Ketjenblack EC-300J with a specific surface area of ~800 m^2^ g^–1^) with a total metal loading of 30 wt%. The powder precursors underwent annealing treatments in 5 vol% H_2_/Ar at 700 °C for 4 h. The average particle size of the obtained PtCo catalysts was analyzed by the Debye–Scherer equation based on the full width at half maximum of X-ray powder diffraction (XRD) patterns and the statistical results from high-angle annular dark-field scanning transmission electron microscopy (HAADF-STEM) images (Fig. 1b and Supplementary Table 1).

We found severe PtCo sintering (XRD size of 14.9 nm) on carbon black supports in the high-temperature H_2_-annealing if no molecule additives were used, which is consistent with reported results of high-loading PtCo IMCs catalyst synthesis^5,6^. When oxygen-containing molecule additives including glucose (GLU), sodium acetate (SAc), or 2,3-benzofuran (BZF) were used for the impregnation synthesis, the XRD size of PtCo was reduced slightly to 8–12 nm. In the cases of nitrogen-containing molecule additives such as 2-acetylpyrrole (APR), sodium glycinate (SGC), and dicyandiamide (DCDA), the XRD size of PtCo was restricted in the range of 5.5–6.5 nm. Notably, sulfhydryl group-containing molecules including 11-mercaptoundecanoic acid (MUA), 2-mercaptobenzimidazole (MBM), 1-mercaptopropane (MPA), and sodium thioglycolate (STG) exhibited outstanding capacity for suppressing PtCo sintering. Especially, the XRD average particle sizes of PtCo were only ~2.5 nm with MPA and STG used as additives in the high-temperature synthesis.

The average particle sizes obtained from the statistics of HAADF-STEM images matched well with the XRD results (Supplementary Fig. 1). We observed a very wide particle size distribution ranging from a few to tens nanometers in the additive-free synthesis; the appearance of abnormally large particles reflected the severe sintering occurred through coalescence and Ostwald ripening^19,38,39^, which was exacerbated for the high-loading catalysts with short inter-particle distance^40,41^. The size distribution was significantly narrowed when using molecule additives; and remarkably, no PtCo particles of >5 nm were found when using MPA or STG as additive (Fig. 1c–f and Supplementary Fig. 2).

### Synthesis of Pt-IMCs catalyst libraries

Considering the anti-sintering capacity and convenience, we chose water-soluble STG as the optimal additive for the synthesis of Pt-IMCs catalyst libraries. We synthesized the Pt-IMCs catalysts by subjecting the carbon black Ketjenblack EC-300J impregnated with metal salt precursors and STG (Pt content of 20–27.7 wt%; *n*_STG_/*n*_Pt_ of 1.5) to the high-temperature H_2_-annealing treatment at 600–1000 °C. To improve the ordering degree, two-step H_2_-annealing protocol was adopted for some Pt-IMCs synthesis^6^. The high-temperature-annealing step was to alloy Pt with enough amount of base metals to approach the stoichiometric ratio of corresponding Pt-IMCs structures and thus to maximize the thermodynamic driving force of disorder-to-order transition; the relatively-low-temperature-annealing step with prolonged time was to make the kinetically controlled atom ordering thoroughly proceed. For each Pt-IMCs synthesis, the temperature of high-temperature-annealing step was optimized to balance the trade-off relations between particle size and ordering degree (Supplementary Table 2). The details of the optimal synthesis recipe for each Pt-IMCs catalyst were summarized in Supplementary Table 3.

**Fig. 2:**
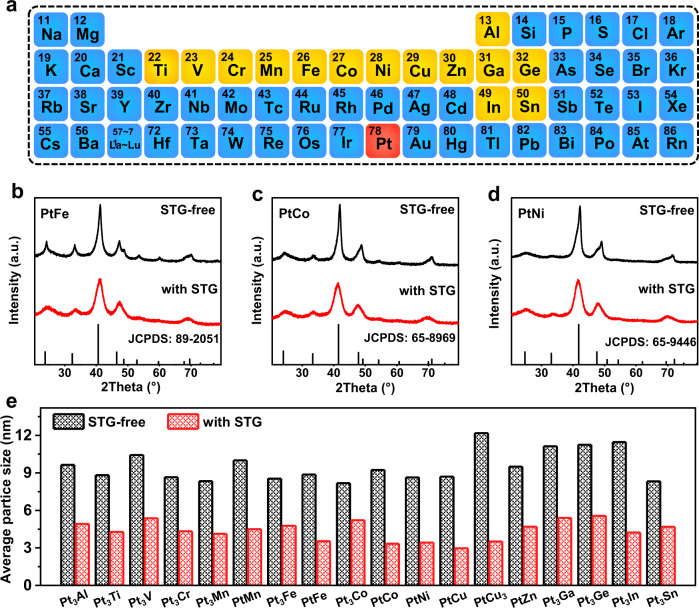
STG-assisted synthesis of Pt-IMCs catalyst libraries. **a** Periodic table showing Pt (highlighted in red) and base metal elements (highlighted in yellow) that constitute Pt-IMCs catalysts libraries. Comparison of the XRD patterns of PtFe (**b**), PtCo (**c**), and PtNi (**d**) prepared with and without STG. **e** Comparison of XRD particle size of the 18 Pt-IMCs catalysts prepared with and without STG.

We totally synthesized 18 binary Pt-IMCs catalysts by the STG-assisted impregnation method, including Pt alloyed with *3d* early transition metals (Pt_3_Ti, Pt_3_V, and Pt_3_Cr), *3d* late transition metals (Pt_3_Mn, PtMn, Pt_3_Fe, PtFe, Pt_3_Co, PtCo, PtNi, PtCu, PtCu_3_, and PtZn), group IIIA metals (Pt_3_Al, Pt_3_Ga, and Pt_3_In), and group IVA metals (Pt_3_Ge and Pt_3_Sn) (Fig. 2a). The Pt-IMCs structures were evidenced by the experimental XRD patterns that showed ordering crystal structure with characteristic superlattice reflection and matched well with the corresponding Joint Committee on Powder Diffraction Standards (JCPDS) cards (Fig. 2b–d and Supplementary Fig. 3). All the Pt_3_M- and PtM_3_-type Pt-IMCs adopted a cubic structure in space group Pm-3m, except for Pt_3_Ge whose space group was I4/mcm; all the PtM-type IMCs adopted a tetragonal structure in space group P4/mmm, except for PtCu whose space group was R-3m. The XRD average particle size of most samples (14 cases) was less than 5 nm, with four cases showing slightly large particle size (5.2–5.6 nm for Pt_3_V, Pt_3_Co, Pt_3_Ga, and Pt_3_Ge) (Fig. 2e and Supplementary Table 2). Contrastively, the XRD size of Pt-IMCs synthesized by the conventional impregnation synthesis (that is, molecule additive-free) was in the range of 8.2–12.2 nm (Fig. 2e).

**Fig. 3:**
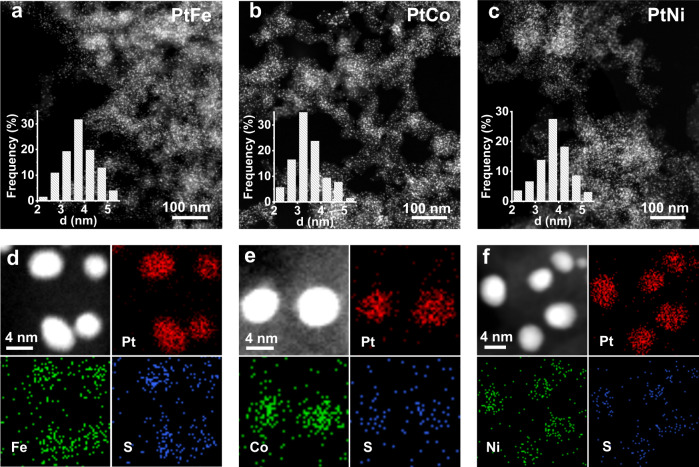
Structural characterization of the Pt-IMCs catalysts. HAADF-STEM images of PtFe (**a**), PtCo (**b**), and PtNi (**c**) prepared with STG. Inserts were the corresponding statistics of particle size distribution. EDS elemental mapping of PtFe (**d**), PtCo (**e**), and PtNi (**f**) prepared with STG.

HAADF-STEM observations confirmed the uniform distribution of the small-sized PtFe, PtCo, and PtNi IMCs nanoparticles on the whole carbon black supports with narrow particle size distributions (Fig. 3a–c). The statistical analyses of particle size based on the HAADF-STEM images were consistent well with the XRD results. Moreover, elemental mapping with energy-dispersive X-ray spectroscopy (EDS) showed that Pt and base metal elements were distributed uniformly in individual particles, and the atomic ratio of Pt/base metal was approximately equivalent to the corresponding stoichiometric value (Fig. 3d–f, Supplementary Figs. 4–8 and Supplementary Table 4).

**Fig. 4:**
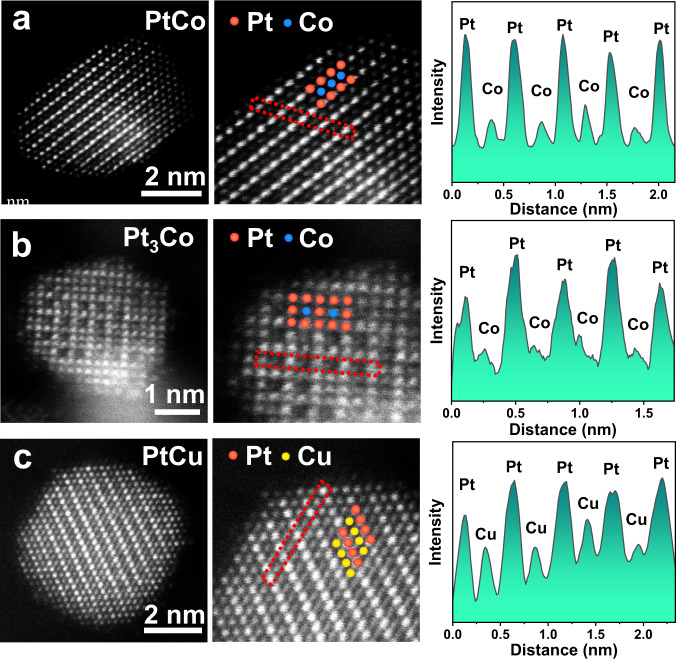
Atomic scale HAADF-STEM characterization of Pt-IMCs. Atomic-resolution HAADF-STEM images and alternative intensity profiles of PtCo (**a**), Pt_3_Co (**b**), and PtCu (**c**). The alternative intensity profiles were obtained from the corresponding region marked by red dashed rectangle.

We used atomic number (Z)-contrast HAADF-STEM imaging to analyze the ordered structures of three representative samples of PtCo, Pt_3_Co, and PtCu on an atomic scale. For PtCo (Fig. 4a), the HAADF-STEM image along [100] direction showed the alternating arrangement of Pt and Co atom columns, represented by the brighter dots and darker dots respectively (Pt columns have a higher intensity than Co columns with a lower Z), indicating the L1_0_-type face-centered tetragonal (*fct*) intermetallic structure. For Pt_3_Co (Fig. 4b), we observed periodic square array of Co columns surrounded by Pt columns at the edges and corners of each unit cell along the [001] zone axis, corresponding to the L1_2_-type face-centered cubic (*fcc*) intermetallic structure. In the case of PtCu (Fig. 4c), the alternating Pt and Cu columns were staggered to align along the [110] direction, which were assigned to the L1_1_-type rhombohedral intermetallic structure. Fast Fourier transform (FFT) patterns (Supplementary Fig. 9) and alternating intensity profiles (Fig. 4) further confirmed the ordered structure of these Pt-IMCs.

The convenience and scalability of the small molecule-assisted impregnation method enabled the grams scale synthesis. We achieved the one-batch synthesis of L1_0_ PtCo catalyst exceeding 6 grams, with a considerable high ordering degree of 65%, a small XRD size of 4.3 nm as well as a narrow particle size distribution revealed by HAADF-STEM observations (Supplementary Fig. 10 and Supplementary Table 5). We also achieved the L1_0_ PtCo catalyst synthesis with a high total metal loading of 45 wt% (Pt content of 34.5 wt%), showing an ordering degree of 56% and a XRD size of 4.7 nm (Supplementary Fig. 11 and Supplementary Table 5).

### Mechanism study

**Fig. 5:**
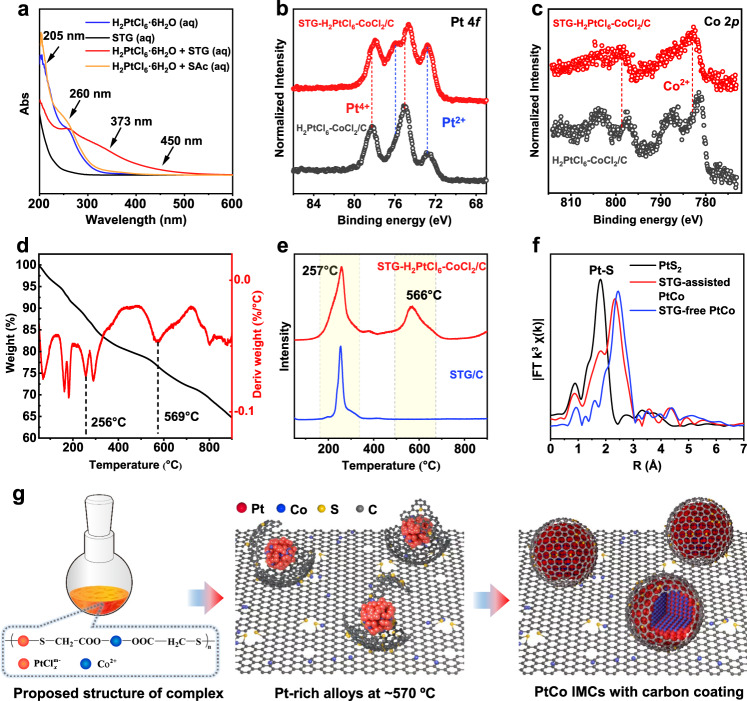
Anti-sintering mechanism. **a** UV–Vis spectra of the H_2_PtCl_6_·6H_2_O solution, STG solution, the H_2_PtCl_6_·6H_2_O/STG solution, and the H_2_PtCl_6_·6H_2_O/SAc solution. XPS results of the Pt 4*f* (**b**) and Co 2*p* (**c**) orbits of the STG-PtCo/C precursor powder and PtCo/C precursor powder. **d** TG result of STG-PtCo/C raw powder from room temperature to 900 °C. **e** MS signal of CH_2_-SH fragment obtained from the STG/C powder and STG-PtCo/C precursor powder. **f** R space of the XAFS results of the standard PtS_2_, and PtCo IMCs catalysts prepared with and without STG. **g** Proposed anti-sintering mechanism of the STG-assisted method.

We performed UV–visible absorption (UV–Vis) spectroscopy, X-ray photoelectron spectroscopy (XPS), thermogravimetry-mass spectrometry (TG-MS), extended X-ray absorption fine structure (EXAFS), HAADF-STEM, and EDS elemental mapping characterizations to understand the anti-sintering mechanism of the small molecule additives, by taking STG-assisted impregnation synthesis of PtCo IMCs as a typical example. We found the discoloration from light yellow to orange when STG was added into the aqueous solution of H_2_PtCl_6_ (Supplementary Fig. 12), indicating the change of coordination structure of platinum. H_2_PtCl_6_ solution showed typical four absorption bands in its UV–Vis spectrum located at around 205, 260, 373, and 450 nm, respectively (Fig. 5a). The absorption bands at 205 and 260 nm were assigned to the ligand-to-metal charge-transfer corresponding to ${}^{1}{{{{{\rm{A}}}}}}_{1g}\to {}^{1}{{{{{\rm{T}}}}}}_{1u}$ transition; the former was due to the transitions from orbitals with ligand σ-character, while the latter was due to the transitions involving orbitals with Cl ligand π-character^42^. The absorption bands at 373 and 450 nm were assigned to the *d*-*d* transitions that reflected the octahedral symmetry of [PtCl_6_]^2−^ configuration^43,44^. We observed significant changes of the intensity of absorption bands at 205, 373, and 450 nm when STG was mixed with H_2_PtCl_6_, whereas no obvious changes of H_2_PtCl_6_ absorption bands were found if replacing STG with SAc that had only carboxyl group. These results suggested that the sulfhydryl group in STG coordinated with platinum by ligand exchanging with Cl^45^.

In high-resolution Pt 4*f* XPS spectra for the H_2_PtCl_6_–CoCl_2_/C, we assigned the peak pair located at 75/78.2 eV and peak pair located at 72.7/76 eV to the Pt (IV) and Pt (II) species, respectively^46^. With the incorporation of STG molecules, we found increased content of Pt (II) species and negative shift of binding energy for Pt (IV) species by ~0.3 eV in STG-H_2_PtCl_6_–CoCl_2_/C, indicating a more electron-enriched sate after ligand exchange (Fig. 5b). Differently, the binding energy of Co 2p showed a positive shift by ~1.4 eV with the incorporation of STG (Fig. 5c). We supposed that the electronic structure change of Co was associated to the coordination of Co (II) with carboxylate group in STG.

TG-MS coupling technique was employed to analyze the thermal decomposition of STG in high-temperature H_2_-annealing. We observed continuous weight loss of STG-H_2_PtCl_6_-CoCl_2_/C during the whole heating process up to 900 °C (Fig. 5d). The collected signal of the characteristic fragment corresponding to CH_2_-SH with the mass-to-charge ratio of 47 appeared at 257 °C and 566 °C, while only one single peak of CH_2_-SH signal at a lower temperature of ~254 °C was found for STG/C (Fig. 5e). Thus, the decomposition peak at the high temperature of 566 °C could assigned to STG coordinated to platinum. Fourier-transformed EXAFS analyses showed a clear trend that the intensity of Pt-Cl/Pt-S bonds gradually decreased as the annealing temperature rising, accompanied by the increased intensity of Pt-Pt/Pt-Co bonds (Supplementary Fig. 13), demonstrating the decomposition of coordination structures and the formation of alloys.

After high-temperature H_2_-annealing of the STG-H_2_PtCl_6_–CoCl_2_/C composite precursor, we observed by high-resolution bright-field STEM that a one-graphene-layer carbon coating with lower contrast almost completely covered the alloy particles and connected with the underlying carbon support (Supplementary Fig. 14). EDS elemental mapping revealed that the majority of residual S elements concentrated on the PtCo particle region (Fig. 3e). Note that the S/Pt atomic ratio has dramatically decreased from 1.5 for the precursor to 0.15 after the annealing (Supplementary Fig. 5). Considering the spatial relationship between S, carbon shells and PtCo particles, we could reasonably infer that S atoms were doped in the carbon layers on the PtCo particle surface. In the Fourier-transformed EXAFS (Fig. 5f), the annealed sample showed an extra shoulder peak similar as the Pt-S bond in PtS_2_, indicating the binding interaction between Pt and doped S atoms in carbon shells. We also characterized several additional STG-assisted Pt intermetallic catalysts including PtFe, PtNi, Pt_3_Ti, and Pt_3_In by high-resolution STEM (Supplementary Fig. 15), the results showed that the carbon shell formation was a general phenomenon in the STG-assisted synthesis.

On the basis of the above systematical characterizations, we propose the anti-sintering mechanism in the STG-assisted synthesis of PtCo IMCs (Fig. 5g). In the initial wet-impregnation step, the sulfhydryl group of STG tended to coordinate with Pt (IV) via the partial ligand exchange with Cl, meanwhile the carboxylate group in STG coordinated with Co (II), to form bimetallic complex network (left panel in Fig. 5g). Upon the annealing, the complex precursors gradually decomposed, along with the release of CH_2_-SH and Cl fragments and the formation of the PtCo bimetallic clusters. The coordinated STG ligands exhibited promoted thermal stability compared to uncoordinated STG as revealed by the TG-MS tests and thus thermally converted to S-doped carbonaceous layers around the PtCo clusters (middle panel in Fig. 5g). Further annealing at the evaluated temperature of 900 °C made the PtCo particles well alloyed with targeted stoichiometric ratio (that is 1:1 for L1_0_ PtCo IMCs). In the final low-temperature-annealing step with prolonged time (600 °C, 6 h), the Pt/Co atoms rearranged into intermetallic structures via a kinetically controlled atom ordering process. Importantly, the physical protective carbon coating and the chemical Pt–S interaction, may function in a synergetic way, to greatly suppress the PtCo sintering during the high-temperature annealing and guaranteed the synthesis of small-sized Pt-IMCs catalysts (right panel in Fig. 5g). The detailed anti-sintering mechanism should differ with various molecule additives, as the capacity for suppressing PtCo sintering was strongly dependent on the molecule structures. But our results demonstrated the general validity of utilizing small molecule additives for the impregnation synthesis of small-sized Pt-IMCs catalysts.

### PEMFCs performance

Six PtM-type IMCs catalysts were selected for the electrochemical measurements, including five L1_0_ structures (PtMn, PtFe, PtCo, PtNi, and PtZn) and one L1_1_ structure (PtCu). Prior to tests, all the catalysts were first oxidized in air (230 °C, 6 h) to gently remove the carbon coating and exposure metal surface evidenced by the STEM images of the samples after air oxidation (Supplementary Fig. 16). EDS mapping demonstrated that the S/Pt atomic ratio was reduced to 0.016 after the oxidation treatment, corresponding to a nearly 10 times of reduction relative to the value of pristine catalyst (0.15). Then the powder underwent acid leaching (0.2 M H_2_SO_4_, 60 °C, 12 h) and low-temperature H_2_-annealing (5 vol% H_2_/Ar, 400 °C, 2 h) treatments successively to form an electrochemically stable and active core/shell structures that were composed of an intermetallic PtCo core and two to three atomic layers of Pt shell (Supplementary Fig. 17)^6,47–49^.

**Fig. 6:**
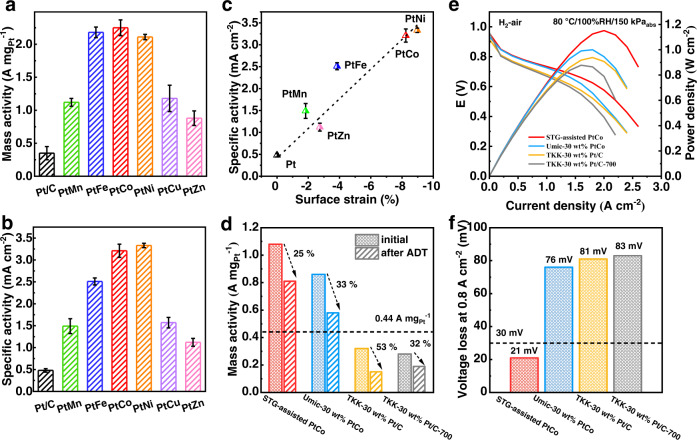
Electrocatalytic performance. RDE mass activity (**a**), and specific activity (**b**) of the STG-assisted Pt-IMCs catalysts and TKK-30 wt% Pt/C. The Pt loading in RDE tests were in the range of 11~13 µg cm^–2^. **c** Correlation between specific activity and calculated surface strain of the five L1_0_ PtM catalysts and Pt/C. The error bars in **a**, **b**, and **c** were obtained after three parallel experiments. **d** Mass activity loss of the MEAs made with cathode catalysts of STG-assisted PtCo, Umic-30 wt% PtCo, TKK-30 wt% Pt/C, and TKK-30 wt% Pt/C-700 after 30,000 cycles’ ADT. **e** H_2_-air polarization curves and power density plots of the MEAs made with cathode catalysts of STG-assisted PtCo, Umic-30 wt% PtCo, TKK-30 wt% Pt/C, and TKK-30 wt% Pt/C-700. For all MEA, the cathode and anode loadings were controlled to be 0.1 and 0.025 mg_Pt_ cm^–2^, respectively. Test conditions: 80 °C, 100% RH, 150 kPa_abs_, outlet H_2_-air at high stoichiometries (H_2_ and air flow rates were fixed at 0.5 and 2.0 L min^–1^, respectively). **f** Voltage loss at 0.8 A cm^–2^ of the MEAs made with cathode catalysts of STG-assisted PtCo, Umic-30 wt% PtCo, TKK-30 wt% Pt/C, and TKK-30 wt% Pt/C-700 after 30,000 cycles’ ADT.

In the rotating disk electrode (RDE) tests, all the six catalysts showed high electrochemically active surface areas (ECSA) of 63.4–86.7 m^2^ g_Pt_^–1^ tested by CO-stripping, which is comparable with that of commercial TKK-30 wt% Pt/C (72.5 m^2^ g_Pt_^–1^) and higher than that of recently reported PtM IMCs catalysts^13,14,35,50^. The PtM IMCs catalysts also showed much higher mass activities of 0.88–2.25 A mg_Pt_^–1^ and specific activities of 1.12–3.33 mA cm^–2^ at 0.9 V_iR-correct_ than those of Pt/C (0.35 A mg_Pt_^–1^ for MA; 0.48 mA cm^–2^ for specific activity) (Fig. 6a, b and Supplementary Table 6). We further observed a strong relationship between ORR activity and surface compressive strain for all the five L1_0_ IMCs catalysts (Fig. 6c and Supplementary Table 7), consistent with our previous results on S-doped carbon supported IMCs catalysts^6^. We predict that further promotion of ORR activity would be realized by shortening the lattice parameter of the intermetallic core to enhance the surface compressive strain.

Finally we chose the STG-assisted PtCo as the cathode catalyst for the PEMFCs tests. The TKK-30 wt% Pt/C, TKK-30 wt% Pt/C annealed at 700 °C for 2 h (named as TKK-30 wt% Pt/C-700), and the Elyst Pt30 0690 catalysts from Umicore (named as Umic-30 wt% PtCo) were also tested as the comparison. The ionomer to carbon (I/C) ratio was optimized for each cathode catalysts (Supplementary Fig. 18). In H_2_-O_2_ single-cell test, STG-assisted PtCo catalyst exhibited a high MA of 1.08 A mg_Pt_^–1^ at 0.9 V, higher than 0.32, 0.28, and 0.86 A mg_Pt_^–1^ for TKK-30 wt% Pt/C, TKK-30 wt% Pt/C-700, and Umic-30 wt% PtCo, respectively, and exceeded the US Department of Energy (DOE) 2025 target of 0.44 A mg_Pt_^–1^. After 30,000 cycles’ ADT, which was performed by a square wave voltage cycling from 0.6 to 0.95 V with a duration of 3 s for each voltage, the MA of the STG-assisted PtCo catalyst declined by 25%, while for TKK-30 wt% Pt/C, TKK-30 wt% Pt/C-700, and Umic-30 wt% PtCo, the MA declined by 53%, 32%, and 33%, respectively (Fig. 6d).

We further tested the performance of STG-assisted PtCo catalyst in H_2_-air single cell. At the test conditions of 80 °C, 100% relative humidity (RH), and 150 kPa_abs_, the current density at 0.8 V for the cathode made of the STG-assisted PtCo catalyst was 412 mA cm^–2^, slightly higher than that of Umic-30 wt% PtCo cathode (395 mA cm^–2^), much higher than that of TKK-30 wt% Pt/C (250 mA cm^–2^) and TKK-30 wt% Pt/C-700 (216 mA cm^–2^), and exceeded the DOE 2025 target of 300 mA cm^–2^ (Fig. 6e and Supplementary Table 8). After 30,000 cycles’ ADT, the voltage loss of the STG-assisted PtCo cathode at 0.8 A cm^–2^ was 21 mV, well meeting the DOE 2025 target (less than 30 mV loss); while for TKK-30 wt% Pt/C, TKK-30 wt% Pt/C-700, and Umic-30 wt% PtCo cathodes, the voltage loss was 83, 81, and 76 mV, respectively (Fig. 6f and Supplementary Fig. 19). H_2_-air single-cell tests were also performed at 94 °C, 65% RH, and 250 kPa_abs_, considering the US DOE target of *Q*/Δ*T* < 1.45 kW °C^–1^ for heat rejection^51,52^. The rated power density of the STG-assisted PtCo cathode at 94 °C reached 1.17 W cm^–2^, much higher than that of TKK-30 wt% Pt/C (0.79 W cm^–2^), TKK-30 wt% Pt/C-700 (0.73 W cm^–2^), and Umic-30 wt% PtCo (0.94 W cm^–2^) cathodes. After 30,000 cycles’ ADT, the rated power density of the STG-assisted PtCo cathode still reached 1.03 W cm^–2^ (Supplementary Fig. 19).

## Discussion

In summary, we developed a small molecule-assisted impregnation method for the general synthesis of small-sized Pt-IMCs catalysts in grams scale with commercial carbon black supports. The molecular additives containing functional groups (particularly sulfhydryl) acted as ligands to coordinate with Pt (IV) in impregnation and then thermally converted into heteroatom-doped graphene layers coating on alloy nanoparticles, which greatly suppressed metal sintering in high-temperature annealing and guaranteed the formation of small-sized Pt-IMCs catalysts. By the molecule-assisted approach, we demonstrated the combinatorial synthesis of intermetallic catalyst libraries consisting of 18 binary Pt-IMCs. The intermetallic libraries were highly mass efficient as low-Pt cathode catalysts in PEMFCs with a high mass activity of 1.08 A mg_Pt_^–1^ and could achieve a high rated power density of 1.17 W cm^–2^. Although we demonstrated the PEMFCs applications in the current work, the Pt intermetallics catalysts library has also great application potentials for other energy-conversion-related electrocatalysis and heterogeneous catalysis, for example, electrocatalytic oxidation^29^, alkane dehydrogenation^53^, and acetylene semi-hydrogenation^54^. Moreover, the combinatorial synthesis of the intermetallic catalysts spanning a wide range of metal elements would allow a comprehensive exploration of a structure-performance relationship for diverse reactions in a systematic manner.

## Methods

### Screening small molecules for suppressing metal sintering

A diversity of small molecules containing coordinating heteroatoms (Fig. 1a) as additives were screened for suppressing metal sintering by taking PtCo for an example in the wet-impregnation synthesis. 0.168 mmol H_2_PtCl_6_·6H_2_O and 0.252 mmol molecule were first dissolved in 30 mL solvent (deionized water for GLU, SAc, APR, SGC, DCDA, and STG; acetone for BZF, MUA, and MBM; hexane for MPA) by stirring in a 100 mL flask for 10 min. Then, 0.218 mmol CoCl_2_·6H_2_O was added and stirred for another 10 min to form a clear mixed solution. 100 mg carbon black (Ketjenblack EC-300J) was added into the above solution and stirred overnight to form a homogeneous dispersion, which was then dried with a rotary evaporator. Finally, the raw powder was annealed at 700 °C for 4 h, followed with naturally cooling down to room temperature. The obtained sample were washed thoroughly by deionized water and then underwent XRD and microscopic characterization to elevate the anti-sintering capacitor of the molecule additives.

### STG-assisted synthesis of Pt-IMCs catalyst libraries

The precursors containing carbon black supports, H_2_PtCl_6_·6H_2_O and base metal salts, as well as the optimal molecule additive (that is, STG) were prepared by the same wet-impregnation procedure described above. The obtained precursors were then subjected to one- or two-step high-temperature H_2_-annealing treatment at 600 to 1000 °C to form Pt-IMCs. For each sample, the annealing temperature was optimized to balance the trade-off between particle size and ordering degree. The data of the optimizing process of the PtCo synthesis was shown in Supplementary Fig. 20 and Supplementary Table 5. Typically, for the optimal synthesis of PtCo IMCs, the precursors composed of 100 mg carbon black, 87 mg H_2_PtCl_6_·6H_2_O (0.168 mmol), 51.9 mg CoCl_2_·6H_2_O (0.218 mmol), and 29 mg STG (0.252 mmol) underwent two-step high-temperature H_2_-annealing treatment (900 °C for 2 h and 600 °C for 6 h in sequence) with a heating/cooling rate of 10 °C min^–1^. The obtained sample were finally washed thoroughly by deionized water. The optimal synthesis recipes for each Pt-IMC catalyst were summarized in Supplementary Table 3. For comparison, Pt-IMCs were also synthesized without molecule additives by the same procedure.

For the grams scale synthesis, the whole synthesis procedure was identical as above, except for expanding the dosage to 8.4 mmol H_2_PtCl_6_·6H_2_O, 12.6 mmol STG, 10.9 mmol CoCl_2_·6H_2_O, 5 g carbon black, and 800 mL deionized water in the wet-impregnation step.

For the synthesis of high-loading PtCo IMCs, the precursors composed of 100 mg carbon black, 166.7 mg H_2_PtCl_6_·6H_2_O (0.322 mmol), 99.5 mg CoCl_2_·6H_2_O (0.418 mmol), and 55.5 mg STG (0.483 mmol) underwent two-step high-temperature H_2_-annealing treatment (750 °C for 2 h and 600 °C for 4 h in sequence) before being washed thoroughly by deionized water. The nominal total metal content was 45 wt% and the nominal Pt content was 34.5 wt%.

### Calculation of the ordering degree

To calculate the ordering degree of different Pt-IMCs samples, we used the ratio of the intensity or integrated area of superlattice reflection to non-characteristic reflections as a quantitative evaluation^19^. For example, for PtCo, we used the ratio of integrated area under (110) plane to the sum of the areas under (111), (200) and (002) planes, *S*_(110)_/(*S*_(111)_ + *S*_(200)_ + *S*_(002)_), as a quantitative evaluation. The value of *S*_(110)_/(*S*_(111)_ + *S*_(200)_ + *S*_(002)_) for the standard *fct*-PtCo (JCPDS: 65-8969) is 0.1677, which corresponds to 100% ordering intermetallic PtCo, then the ratio of *S*_(110)_/(*S*_(111)_ + *S*_(200)_ + *S*_(002)_) calculated from PtCo prepared with STG to 0.1677 is the corresponding ordering degree.

### Calculation of the surface strain of L1_0_ PtM catalysts

The surface strain was calculated based on the area change of intermetallic structure relative to bulk Pt. The areas of the triangles shown in Supplementary Fig. 21 can be calculated by the Heron’s formula as follows:

2 $$p=1/2({l}_{\langle 110\rangle }+{l}_{\langle 101\rangle }+{l}_{\langle 011\rangle })$$

3 $$S=\sqrt{p(p-{l}_{\langle 110\rangle })(p-{l}_{\langle 101\rangle })(p-{l}_{\langle 011\rangle })}$$

4 $${{{{{\rm{Surface}}}}}}\,{{{{{\rm{strain}}}}}}=\frac{{S}_{{{{{\rm{IMC}}}}}}-{S}_{{{{{\rm{bulkPt}}}}}}}{{S}_{{{{{\rm{bulkPt}}}}}}}\times 100\%$$

where ${l}_{\langle 110\rangle }$, ${l}_{\langle 101\rangle }$, and ${l}_{\langle 011\rangle }$ represent the length of three triangle sides (*l* = 2 × *d*_Pt-Pt_ or *d*_Pt-M_). The calculated surface strain for all the *fct* intermetallic catalysts was summarized in Supplementary Table 7.

### Characterization

XRD patterns were obtained on a Japan Rigaku DMax-γA rotating anode X-ray diffractometer using Cu K-α radiation (*λ* = 1.54056 Å) at 40 kV and 150 mA. UV–Vis spectra were measured on a UV-3600i plus spectrometer. XPS measurement was carried out on an X-ray photoelectron spectrometer (ESCALab MKII) with an excitation source of Mg Kα radiation (1253.6 eV). TG-MS were measured with TGA-8000 thermogravimetry and Clarus SQ 8Tmass spectrometer. Atomic-resolution HAADF-STEM images and high-resolution bright-field STEM images were taken by a JEM-ARM 200 F Atomic Resolution Analytical Microscope operating at 200 kV. Low-magnification HAADF-STEM images and EDS mapping images were obtained on FEI Talos F200X, equipped with Super X-EDS system at 200 kV. Inductively coupled plasma atomic emission spectrometry (ICP-AES) measurements were carried out on a Thermo Scientific iCAP 7400. X-ray absorption spectra were measured at the beamline 1W1B station of the Beijing Synchrotron Radiation Facility (BSRF) operated at 2.5 GeV and 250 mA.

### RDE measurements

Five L1_0_ catalysts (PtMn, PtFe, PtCo, PtNi, and PtZn) and one L1_1_ catalyst (PtCu) were selected for the electrocatalytic studies. Prior to electrocatalytic tests, all the catalysts were treated successively by air oxidization (230 °C, 6 h), acid leaching (0.2 M H_2_SO_4_, 60 °C, 12 h), and low-temperature H_2_-annealing (5 vol.% H_2_/Ar, 400 °C, 2 h) to remove carbon coating and to form active and stable Pt-IMCs@Pt core/shell structures. The nominal total metal loading of the pristine catalysts was 30 wt%. The exact Pt contents of the treated catalysts used for electrochemical measurements were quantified by ICP-AES to be 25.7 wt% for PtMn, 24.6 wt% for PtFe, 24.5 wt% for PtCo, 23.5 wt% for PtNi, 24.3 wt% for PtCu, and 22.1 wt% for PtZn.

RDE measurements were performed by using a CHI Electrochemical Station (Model 760E) in a three-electrode electrochemical cell at room temperature. Hg/Hg_2_SO_4_ was used as a reference electrode and Pt foil was used as a counter electrode. The reference electrode potentials in this work were calibrated to the reversible hydrogen electrode (RHE) potentials under hydrogen-saturated 0.1 M HClO_4_ solution for each test. The catalyst ink was prepared by sonicating the mixture consisting of 4 mg catalyst and 40 µL (5 wt%) Nafion in 2 mL isopropanol. Then, the ink was drop-coated onto the working electrode (a glassy carbon disk with diameter 5.0 mm) with Pt loading of 11~13 µg cm^–2^ and dried at room temperature in air. After drying, the catalysts were electrochemically activated by cyclic voltammetry (CV) scanning between 0.05 and 1.05 V at 250 mV s^–1^ in N_2_-saturated 0.1 M HClO_4_ solution (around 200 cycles). Then the linear scanning voltammetry (LSV) curves were obtained from 0.05 V to 1.05 V at 10 mV s^–1^ in O_2_-saturated 0.1 M HClO_4_ with the rotating speed at 1600 rpm (Supplementary Fig. 22).

The mass related kinetic current density (*J*_k_) of catalyst at 0.9 V vs. RHE was calculated according to: *J*_k_ = *J* × *J*_L_/((*J*_L_ − *J*) × *L*_Pt_), where *J*, *J*_L_, and *L*_Pt_ represent the current density at 0.9 V vs. RHE, the diffusion limited current density and Pt loading, respectively. Capacitance-correction and IR-correction were performed for the MA calculation on the WaveDriver 20 bipotentiostat (Pine Instrument Company, USA) in 0.1 M HClO_4_ solution from 0.1 Hz to 10,000 Hz with an amplitude of 5 mV, the initial potential was set as 0.05 V.

The electrochemical active surface area (ECSA) was calculated from CO-stripping curve (Supplementary Fig. 23). CO-stripping test was conducted by first bubbling CO into 0.1 M HClO_4_ electrolyte and holding potential at 0.05 V for 30 min, followed by bubbling N_2_ into the electrolyte for 30 min. Then CV curve was collected by scanning from 0.05 V to 1.05 V at a scanning rate of 20 mV s^–1^. The ECSA was calculated according to: ECSA = (*S*_CO_/*V*)/(0.42 × *L*_Pt_). *S*_CO_, *V*, and *L*_Pt_ represent the integration area of CO desorption, sweep speed, and Pt loading, respectively.

### PEMFCs tests

The cathode catalyst ink was made by mixing the TKK-30 wt% Pt/C, TKK-30 wt% Pt/C-700, Umic-30 wt% PtCo, or treated STG-assisted PtCo catalyst with the ionomer solution (D2020® perfluorosulfonic acid ionomer with an equivalent weight of 950 g mol^–1^) and a water-n-propanol solvent mixture (the water to n-propanol ratio was 1), followed by sonicating the dispersion in ice water for 1.5 h. The I/C ratio ranging from 0.6 to 0.9 for the four catalysts was screened to obtain the optimal performance. The optimization process showed that the MEAs made with cathode catalysts of TKK-30 wt% Pt/C, TKK-30 wt% Pt/C-700, and STG-assisted PtCo exhibited the optimal performance with the I/C ratio of 0.8, while the Umic-30 wt% PtCo cathode exhibited the optimal performance with the I/C of 0.9 (Supplementary Fig. 18). The preparation of the anode catalyst ink using TKK-30 wt% Pt/C was similar with the cathode ink except for setting the I/C ratio to 0.8. The concentrations of cathode and anode catalyst inks were all 5 mg mL^–1^. The catalyst-coated-membrane (CCM) with an active area of 5 cm^2^ was prepared on Nafion membrane (GORE, ~12 μm) with an ultrasonic spray equipment (Siansonic UC320, Siansonic Technology Co., Ltd). The fabricated CCM was dried to completely evaporate the solvents. A gas diffusion layer (GDL) including a microporous layer was used (Freudenberg H24CX483) with a thickness of 230 μm. Two GDLs, two gaskets (140 μm), and the prepared CCM were pressed to obtain the membrane electrode assembly (MEA). The Pt loadings of all MEAs at cathode and anode were controlled to be 0.1 and 0.025 mg_Pt_ cm^–2^, respectively.

The seven channel serpentine flow field was applied for the all single-cell tests. The pressure drop between the inlet and outlet of the flow filed was negligible. The MEAs were tested in the Scribner 850e fuel cell test stand linked with a Scribner 885 potentiostat at 80/94 °C, 150/250 kPa_abs_ and 100/65% RH.

For the H_2_–O_2_ single-cell tests, the cell temperature was maintained at 80 °C. The backpressure was set at 50 kPa to compensate the steam pressure so that the net pressure of H_2_ or O_2_ was maintained at 100 kPa. Prior to the data collection, an activation was applied by scanning the voltage from open circuit voltage to 0.2 V, and then performing a voltage recovery (VR) process in the condition of 40 °C, 150% RH, 150 kPa_abs_ with H_2_/air flow at a constant voltage of 0.2 V for 2~4 h, until a stable polarization curve was obtained^55^. The MA was collected at 0.9 *V*_iR-correct_, 80 °C, 100% RH, 150 kPa_abs_ H_2_-O_2_ at flow rates of 0.2/0.5 L min^–1^. The fuel cell was operated at 0.9 *V*_iR-correct_ for 15 min and the MA was based on the average current density at the last 1 min.

For the H_2_-air single-cell tests, the test conditions were 80 °C/100% RH/150 kPa_abs_ or 94 °C/65% RH/250 kPa_abs_. H_2_ and air flow rates were fixed at 0.5 and 2 L min^–1^, respectively. The ADT test was performed using a square wave voltage from 0.6 to 0.95 V with a duration of 3 s for each voltage level, and run up to 30,000 cycles at 80 °C, atmosphere pressure, 100% RH, with H_2_/N_2_ flow of 0.1/0.075 L min^–1^ for the anode and cathode, respectively.

## Supplementary information

Supplementary Information

## Data Availability

The additional XRD patterns, STEM images, XAFS results, catalysis performance, and ChemDraw structures data generated in this study are provided in the Supplementary Information/Source Data file.
