# Supplementary material for: Small molecule-assisted synthesis of carbon supported platinum intermetallic fuel cell catalysts
Source: Nat Commun. 2022 Oct 31;13:6521. doi: 10.1038/s41467-022-34037-7 (PMC9622856; doi:10.1038/s41467-022-34037-7)
Supplement: Supplementary file 1 — Supplementary Information [file 41467_2022_34037_MOESM1_ESM.pdf]

Supplementary Materials

Small molecule-assisted synthesis of carbon supported platinum intermetallic fuel cell catalysts

Tian-Wei Song[1], Cong Xu[1], Zhu-Tao Sheng[2], Hui-Kun Yan[1], Lei Tong[1], Jun Liu[3,4], Wei-Jie Zeng[1], Lu-Jie Zuo[1], Peng Yin[1], Ming Zuo[1], Sheng-Qi Chu[5], Ping Chen[6], Hai-Wei Liang[1]*

[1]Hefei National Research Center for Physical Sciences at the Microscale, Department of Chemistry, University of Science and Technology of China, Hefei 230026, China

[2]College of Chemistry and Materials Science, Anhui Normal University, Wuhu 241000, China

[3]Institute of Solid State Physics, Hefei Institutes of Physical Science, Chinese Academy of Sciences, Hefei 230031, China

[4]Anhui Contango New Energy Technology Co., Ltd, Hefei 230088, China

[5]Institute of High Energy Physics, Chinese Academy of Sciences, Beijing 100049, China

[6]School of Chemistry and Chemical Engineering, Anhui University, Hefei 230601, China

*email: hwliang@ustc.edu.cn

Chemicals and materials

Vanadium trichloride (VCl_3, 97%) and perchloric acid ($HClO_4$, 70%) were purchased from Sigma-Aldrich. Germanium chloride ($GeCl_4$, 99.9999%) were purchased from Alfa Aesar. Dicyandiamide (DCDA, 99%) was purchased from J&K Scientific Ltd. Indium chloride ($InCl_3$, 99.9%), gallium chloride ($GaCl_3$, 99.999%), 2,3-Benzofuran (99%), glucose (98%), 2-acetylpyrrole (99%), 11-Mercaptoundecanoic acid (95%), and 2-Mercaptobenzimidazole (98%) were purchased from Shanghai Macklin Biochemical Co., Ltd., China. All other chemicals were purchased from Sinopharm Chemical Reagent Co. Ltd., China, including hexachloroplatinic hexahydrate ($H_2PtCl_6{\cdot}6H_2O$, 99%), aluminium chloride ($AlCl_3{\cdot}6H_2O$, 97%), titanium tetrachloride ($TiCl_4$, 98%), chromium chloride hexahydrate ($CrCl_3{\cdot}6H_2O$, 99%), manganous chloride tetrahydrate ($MnCl_2{\cdot}4H_2O$, 99%), ferric chloride hexahydrate ($FeCl_3$, 97%), cobalt chloride hexahydrate ($CoCl_2{\cdot}6H_2O$, 99%), nickel chloride hexahydrate ($NiCl_2{\cdot}6H_2O$, 98%), copper nitrate trihydrate ($Cu(NO_3)_2{\cdot}3H_2O$, 99%), zinc nitrate hexahydrate ($Zn(NO_3)_2{\cdot}6H_2O$, 99%), stannous chloride dihydrate ($SnCl_2{\cdot}2H_2O$, 98%), sodium acetate (99.99%), sodium glycinate (98%), 1-mercaptopropane (99.5%), sodium thioglycolate (97%), and sulfuric acid (H_2SO_4, 95%~98%). All the chemicals were used as received without further purification. Deionized water (18.2 MΩ/cm) used in all experiments was prepared by passing through an ultra-pure purification system.

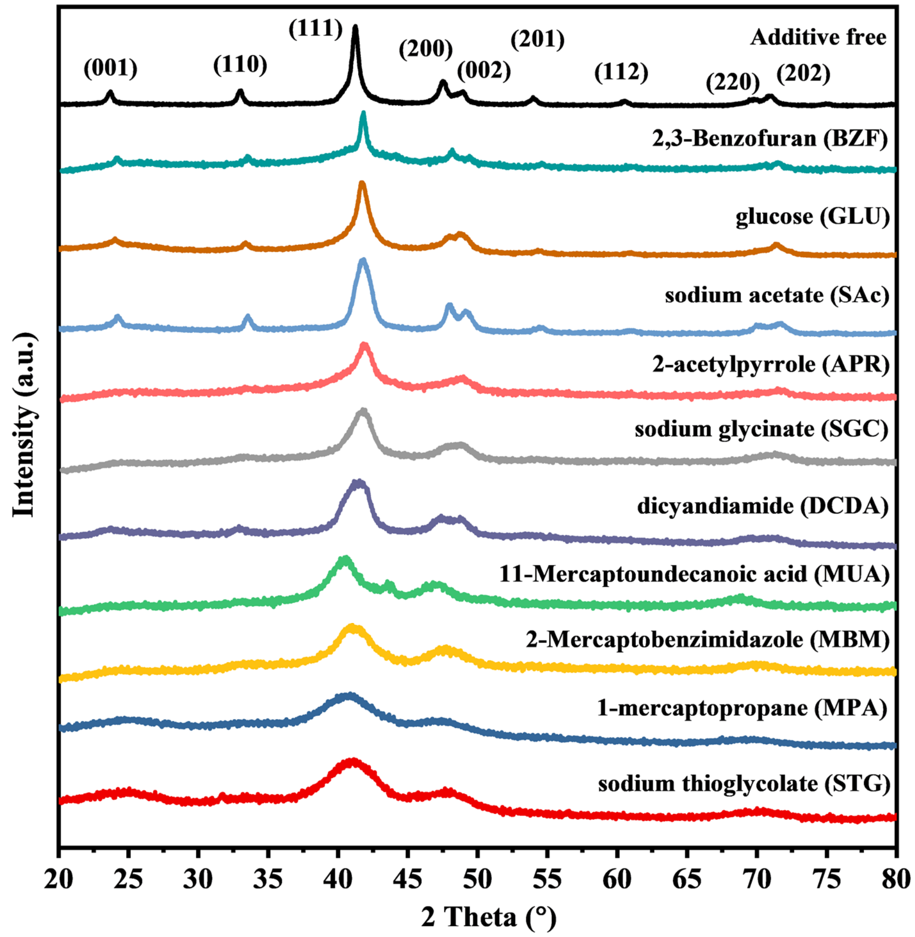

Supplementary Figure 1. XRD patterns of the PtCo catalysts prepared with different molecule additives. The total metal loading of all the samples were controlled to be 30 wt%.

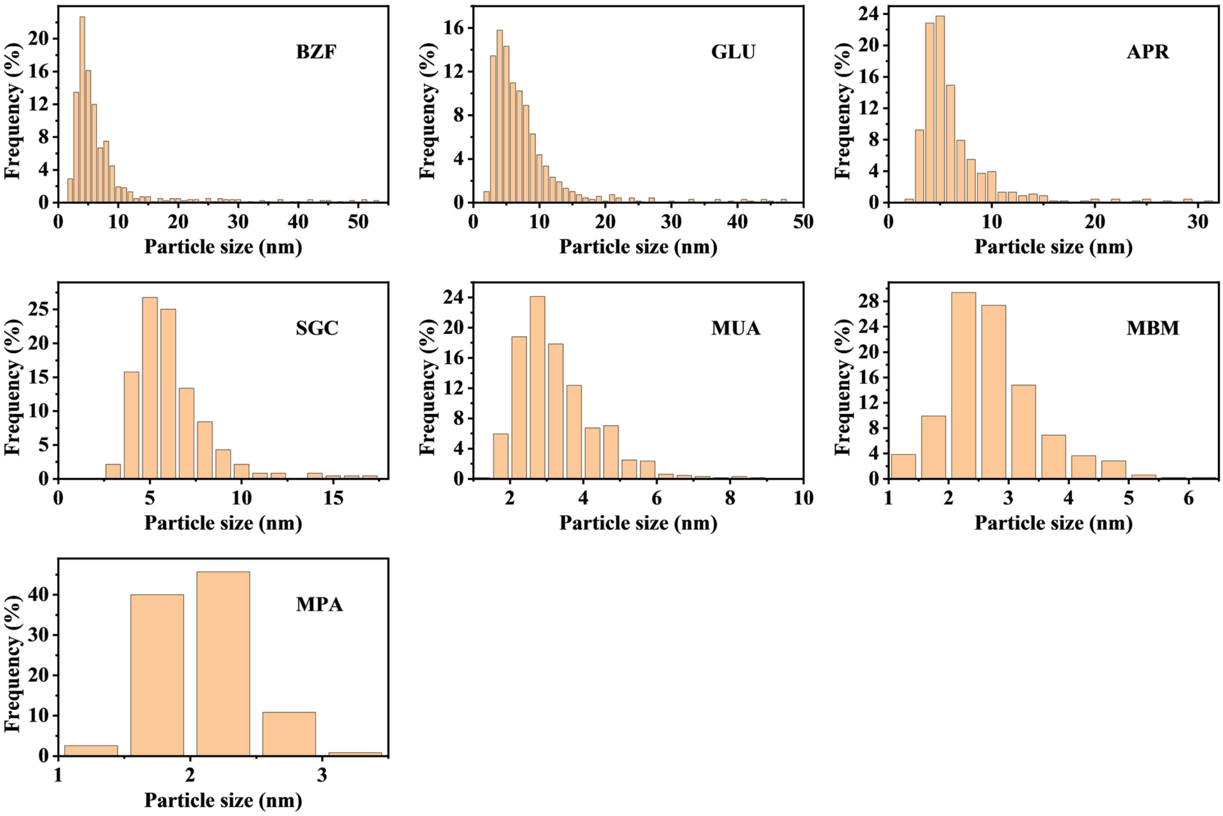

Supplementary Figure 2. Particle size distribution histograms of the PtCo catalysts prepared with different molecule additives, which were obtained through statistic of more than 500 particles in HAADF-STEM images.

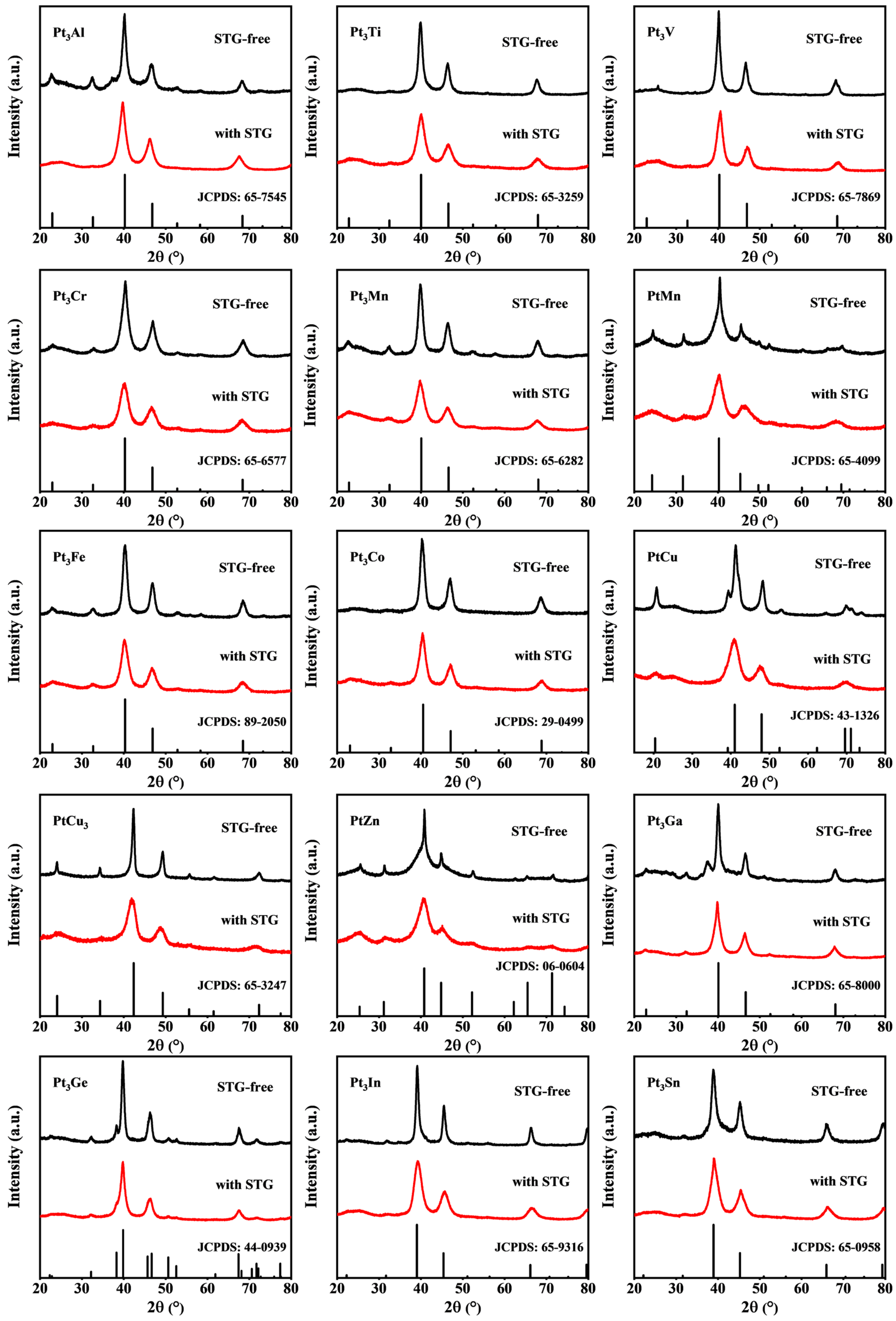

Supplementary Figure 3. XRD patterns of the Pt-IMCs catalysts prepared with and without STG. The JCPDS cards of the corresponding IMCs structures were also shown.

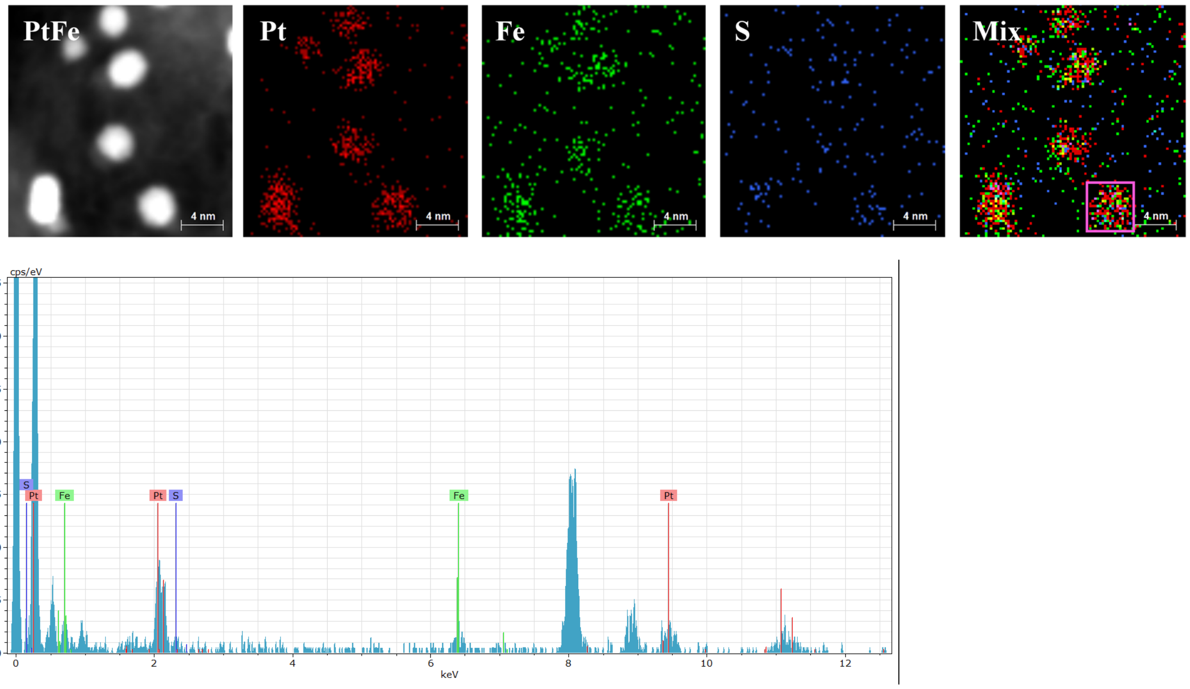

Supplementary Figure 4. EDS elemental mapping and EDS spectra of the PtFe IMCs catalyst.

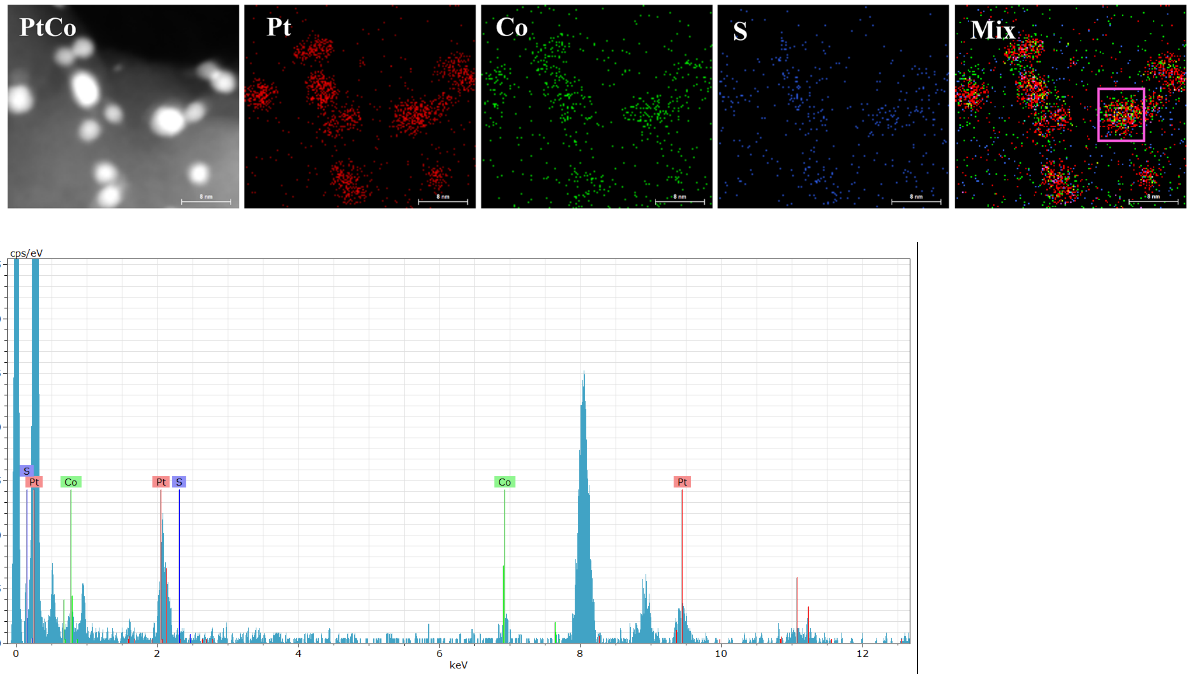

Supplementary Figure 5. EDS elemental mapping and EDS spectra of the PtCo IMCs catalyst.

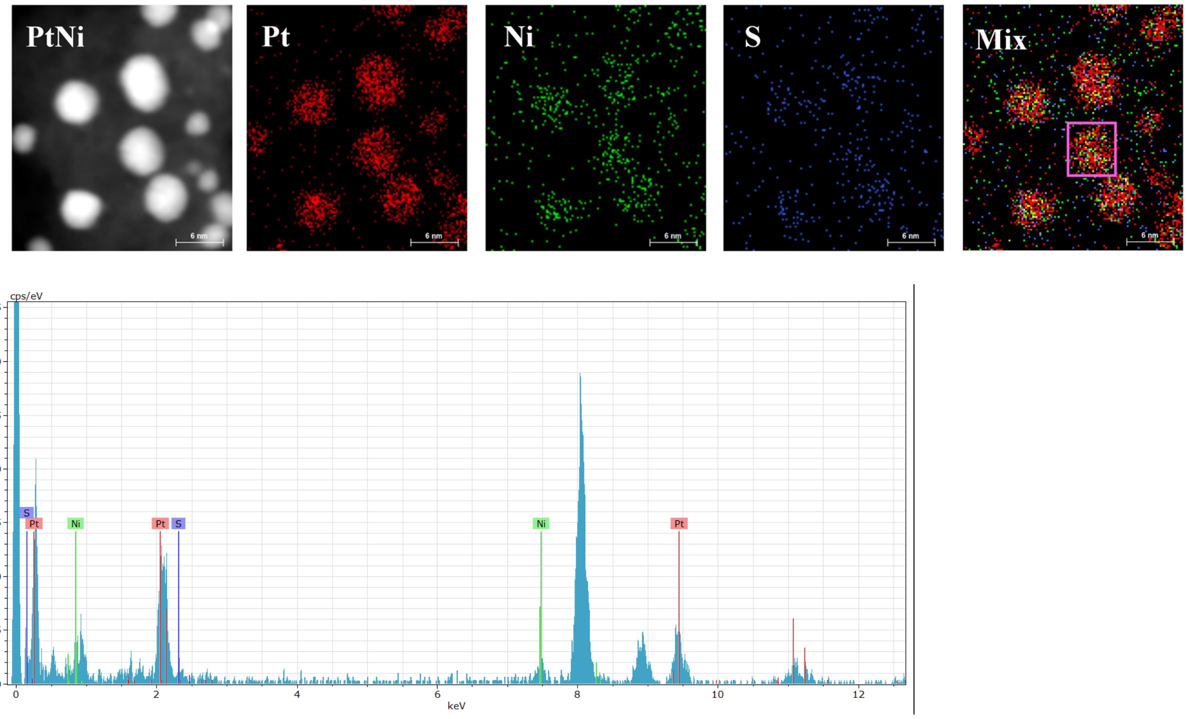

Supplementary Figure 6. EDS elemental mapping and EDS spectra of the PtNi IMCs catalyst.

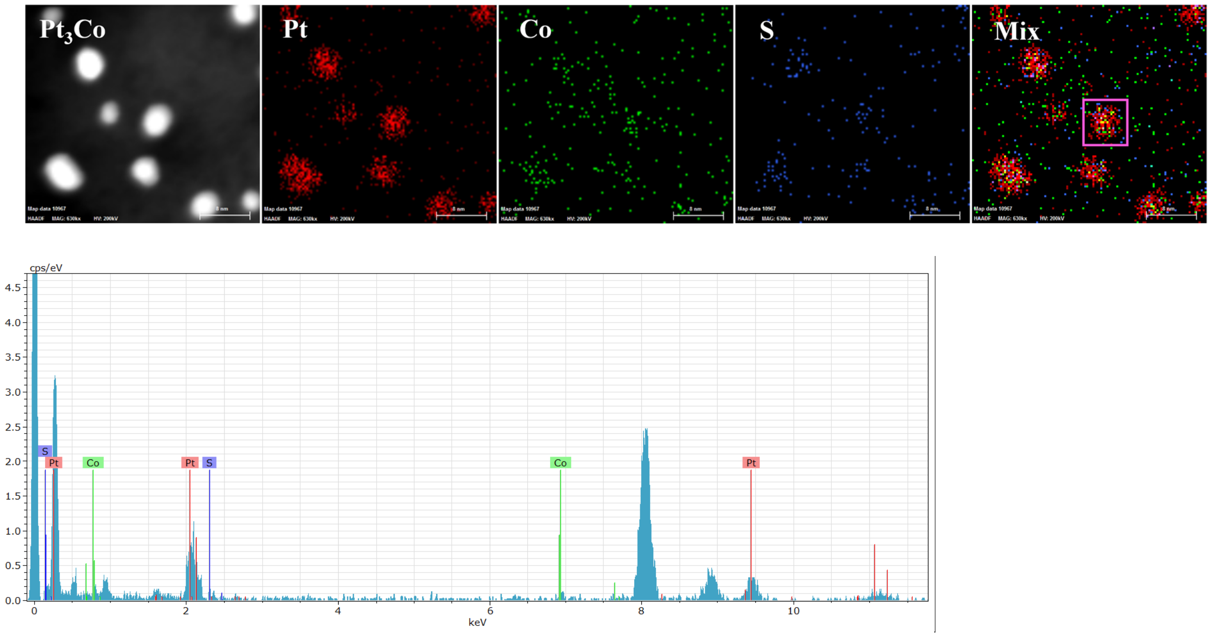

Supplementary Figure 7. EDS elemental mapping and EDS spectra of the Pt_3Co IMCs catalyst.

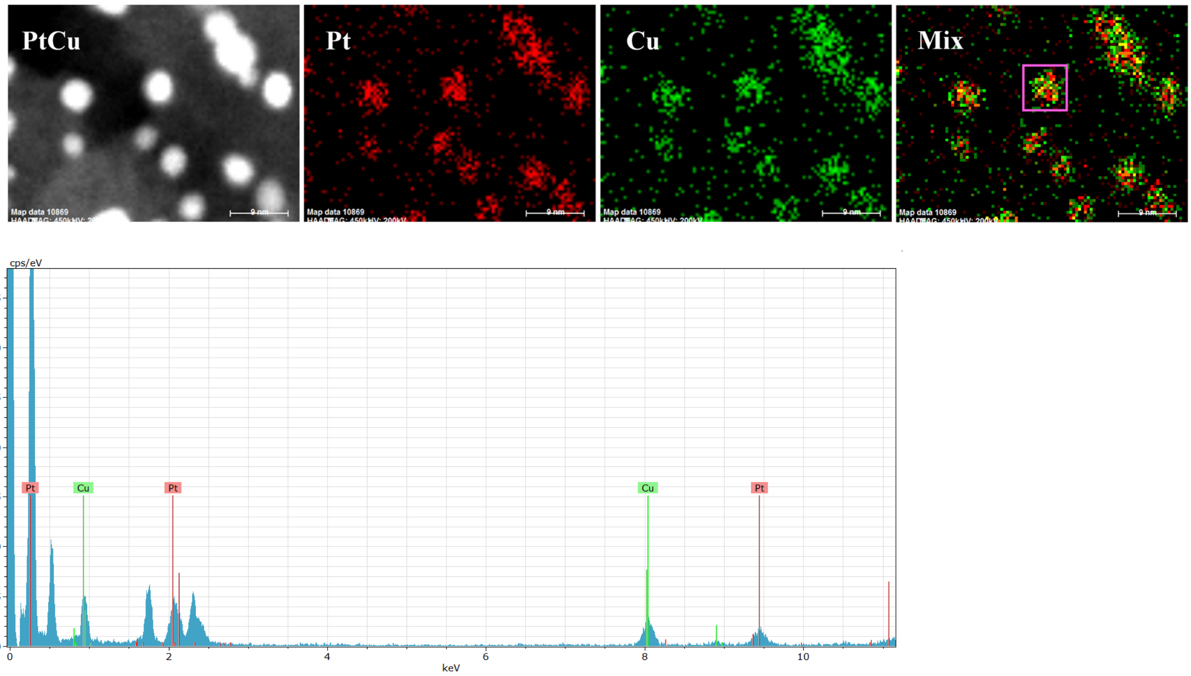

Supplementary Figure 8. EDS elemental mapping and EDS spectra of the PtCu IMCs catalyst. Molybdenum microgrid was used instead of cooper microgrid for the EDS measurements.

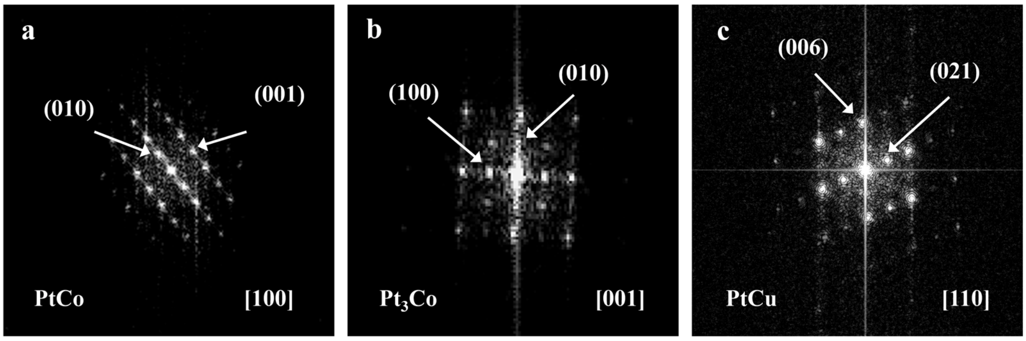

Supplementary Figure 9. FFT patterns of PtCo (a), Pt_3Co (b), and PtCu (c) catalysts whose atomic-resolution HAADF-STEM images were shown in Fig 4.

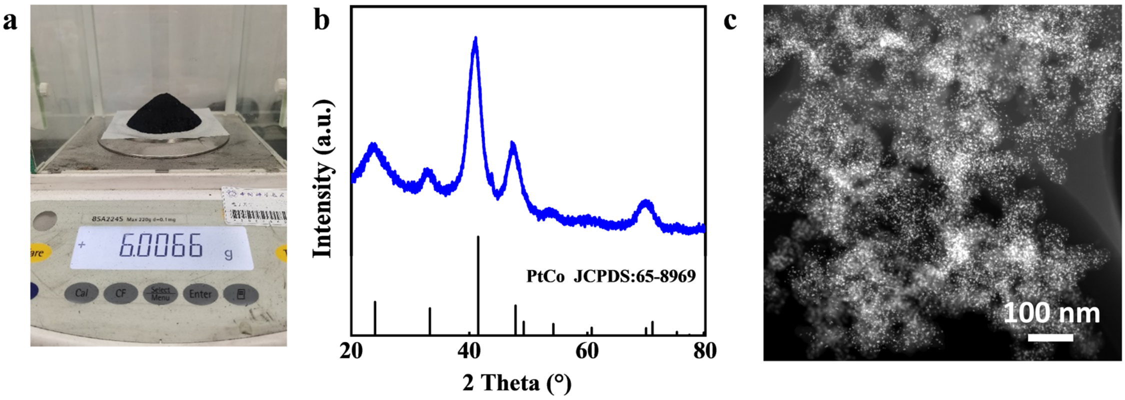

Supplementary Figure 10. (a) Photograph showing the large-scale synthesis of the PtCo IMCs catalyst with STG as additives, the one-batch production could exceeded 6 grams. (b) XRD patterns and (c) HAADF-STEM image of the PtCo IMCs catalyst synthesized in grams scale.

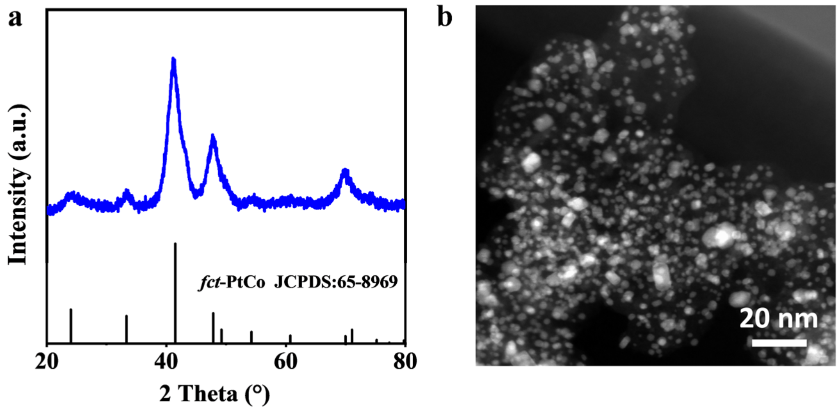

Supplementary Figure 11. (a) XRD patterns and (b) HAADF-STEM image of 45 wt% PtCo prepared with STG additives.

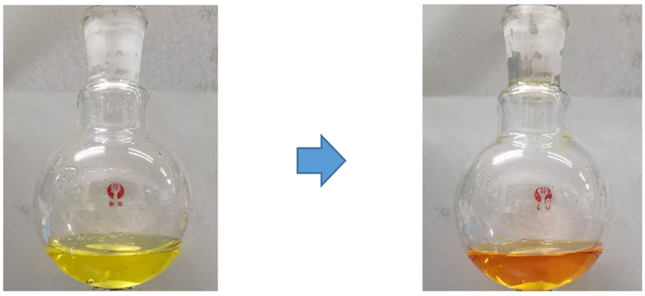

$H_2PtCl_6{\cdot}6H_2O$ (aq) $H_2PtCl_6{\cdot}6H_2O$ (aq) + STG

Supplementary Figure 12. Photographs showing the discoloration of $H_2PtCl_6{\cdot}6H_2O$ solution when adding STG.

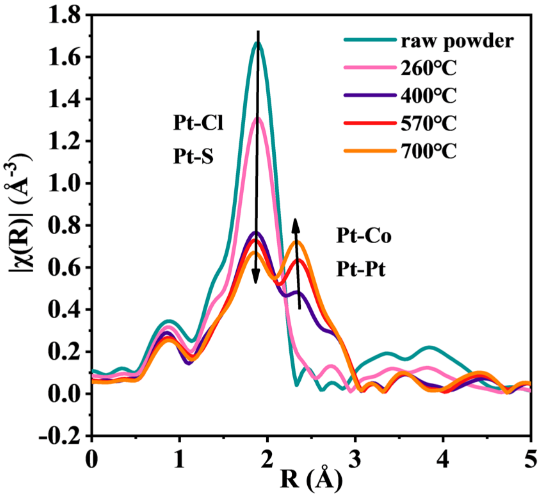

Supplementary Figure 13. R space of the XAFS results for the samples obtained after annealing of STG-H_2PtCl_6-$CoCl_2$/C precursor at different temperatures for 10 min.

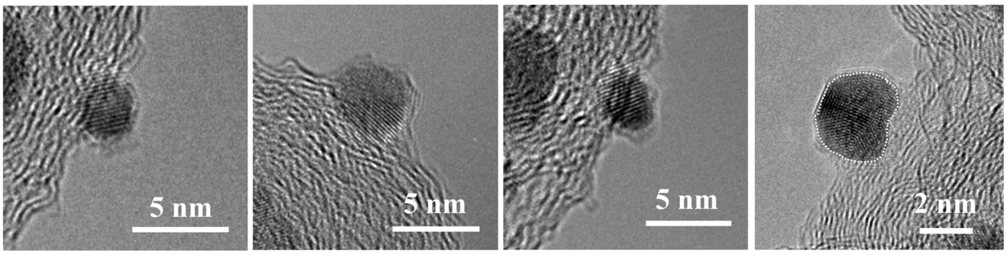

Supplementary Figure 14. High-resolution bright-field STEM images of the PtCo IMCs catalyst prepared with STG additives, showing the formation of thin carbon coating around PtCo particle.

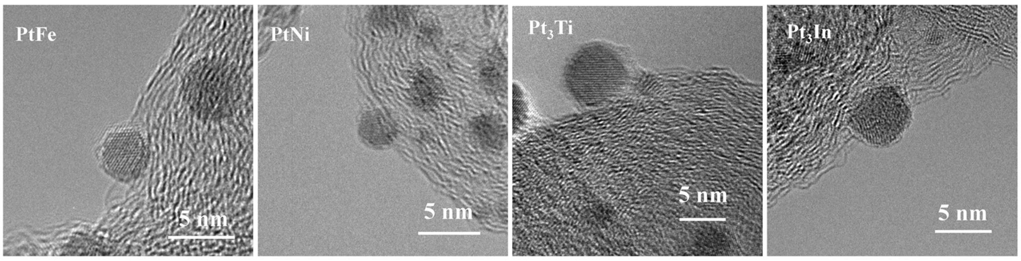

Supplementary Figure 15. High-resolution bright-field STEM images of PtFe, PtNi, Pt_3Ti, and Pt_3In IMCs catalyst prepared with STG additives.

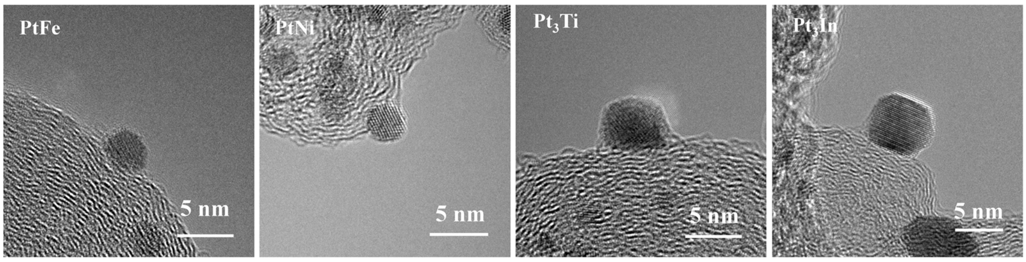

Supplementary Figure 16. High-resolution bright-field STEM images of STG-assisted PtFe, PtNi, Pt_3Ti, and Pt_3In IMCs catalyst after air oxidation.

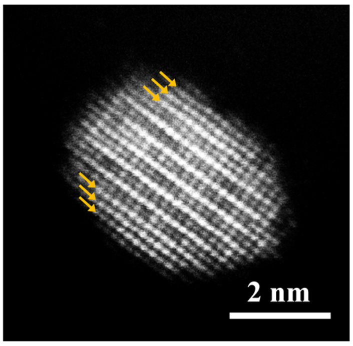

Supplementary Figure 17. Atomic-resolution HAADF-STEM image of the post-treated PtCo IMCs catalyst that was composed of an intermetallic PtCo core and two to three atomic layers of Pt shell.

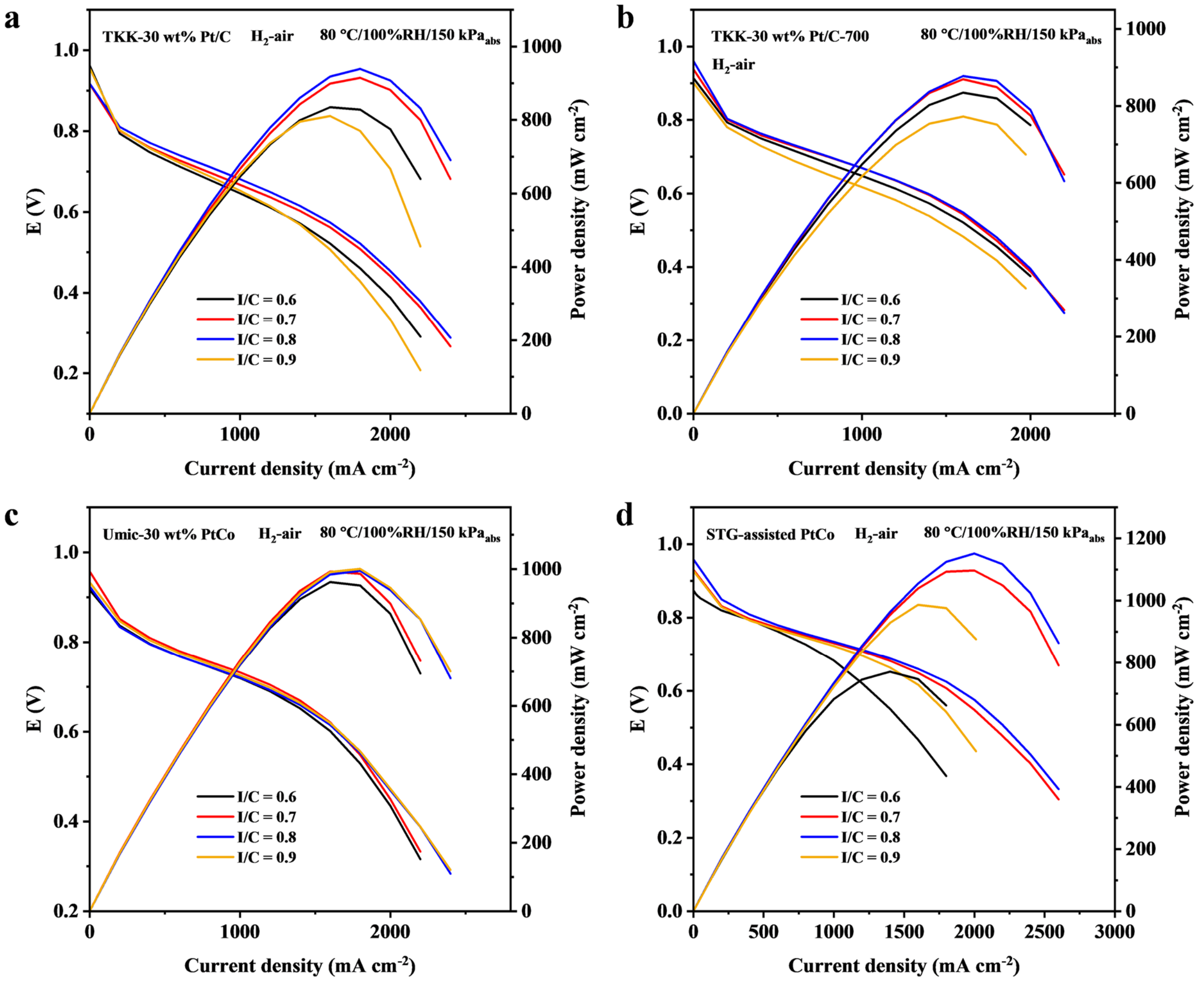

Supplementary Figure 18. The I/C ratio optimization process of TKK-30 wt% Pt/C (a), TKK-30 wt% Pt/C-700 (b), Umic-30 wt% PtCo (c), and STG-assisted PtCo (d) catalysts. The I/C ratio ranged from 0.6 to 0.9, and the MEAs made with catalysts of TKK-30 wt% Pt/C, TKK-30 wt% Pt/C-700, and STG-assisted PtCo exhibited the optimal performance with the I/C ratio of 0.8, while the Umic-30 wt% PtCo exhibited the optimal performance when the I/C is 0.9.

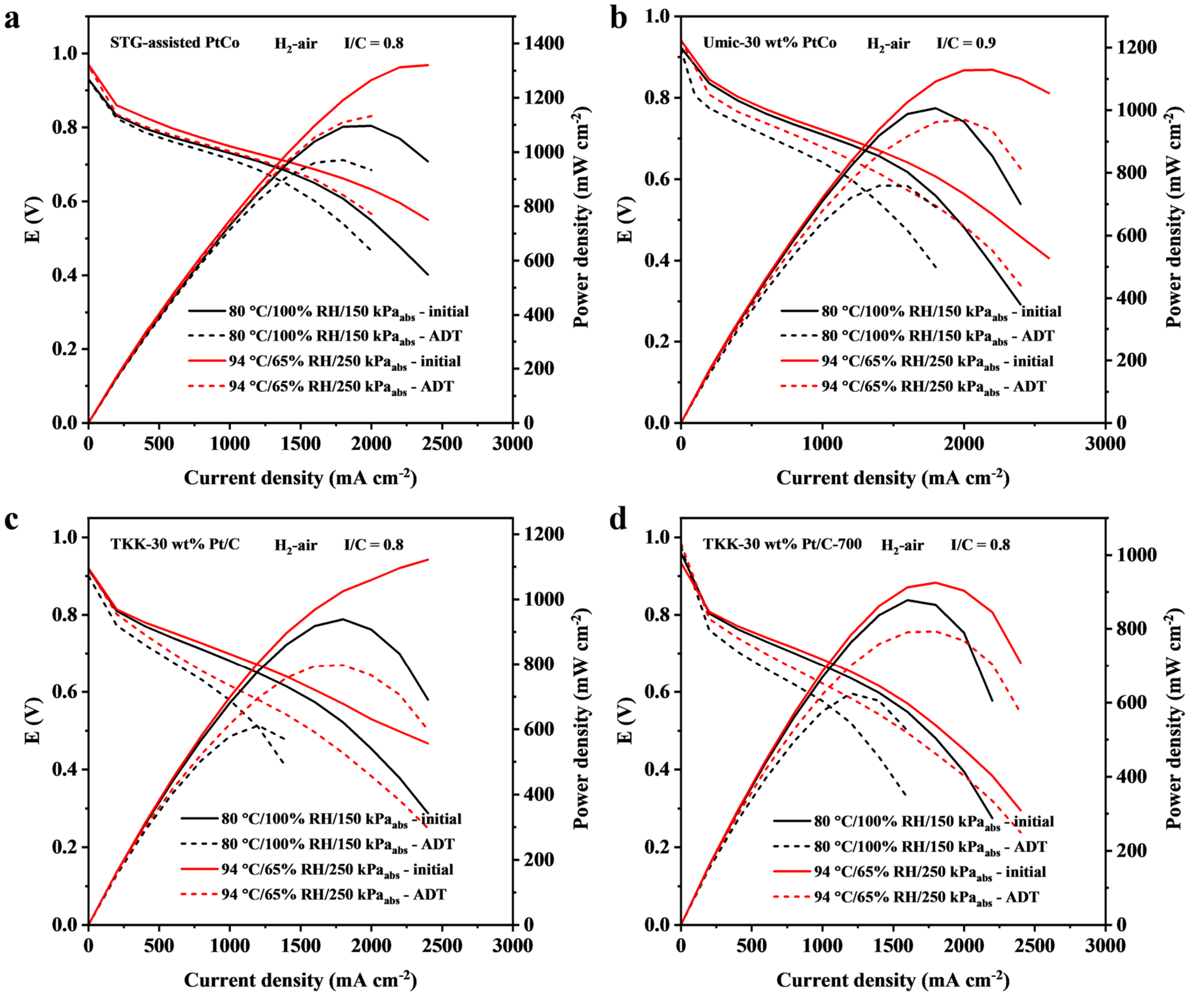

Supplementary Figure 19. H_2-air polarization curves and power density plots of STG-assisted PtCo (a), Umic-30 wt% PtCo (b), TKK-30 wt% Pt/C (c), and TKK-30 wt% Pt/C-700 (d) at the beginning and after 30,000 cycles' ADT in the single-cell tests. All the catalysts used the optimal I/C ratio. Test conditions: 80 °C, 100% RH, 150 kPa_{abs}, or 94 °C, 65% RH, 250 kPa_{abs}.

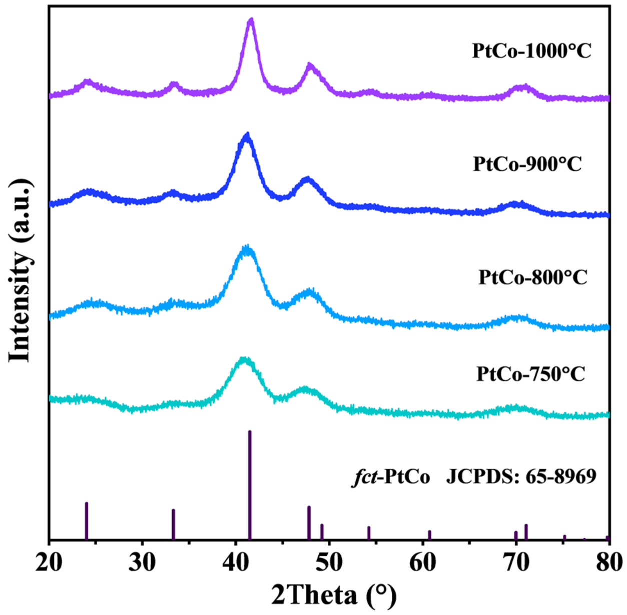

Supplementary Figure 20. XRD patterns of the PtCo catalysts prepared with STG additive at different temperatures, showing the gradually increased average particle size and ordering degree with temperature.

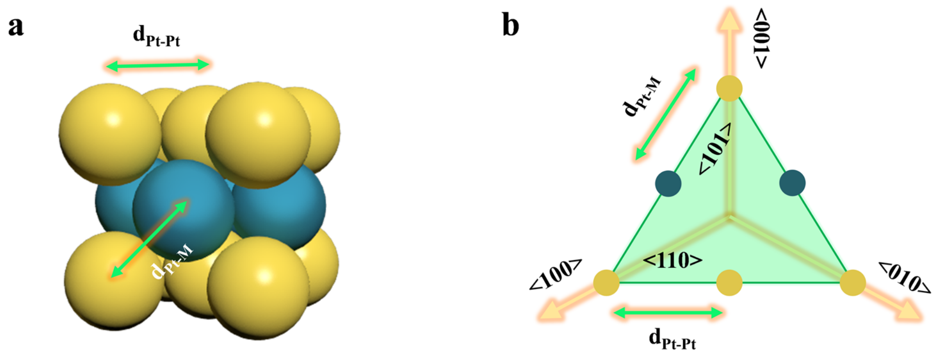

Supplementary Figure 21. (a) Crystal structure of *fct*-PtM, yellow balls represent Pt, blue balls correspond M (M is Mn, Fe, Co, Ni, or Zn). (b) Schematic illustration of lattice mismatch of (111) facet, yellow circles represent Pt and blue circles represent M.

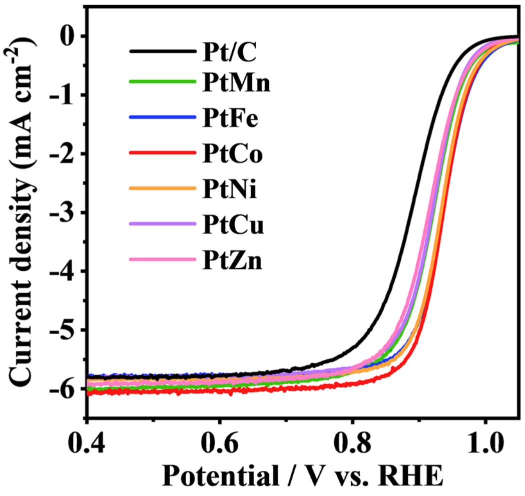

Supplementary Figure 22. RDE polarization curves of the five $L1_0$ PtM IMCs, the $L1_1$ PtCu IMCs, and the commercial TKK-30 wt% Pt/C.

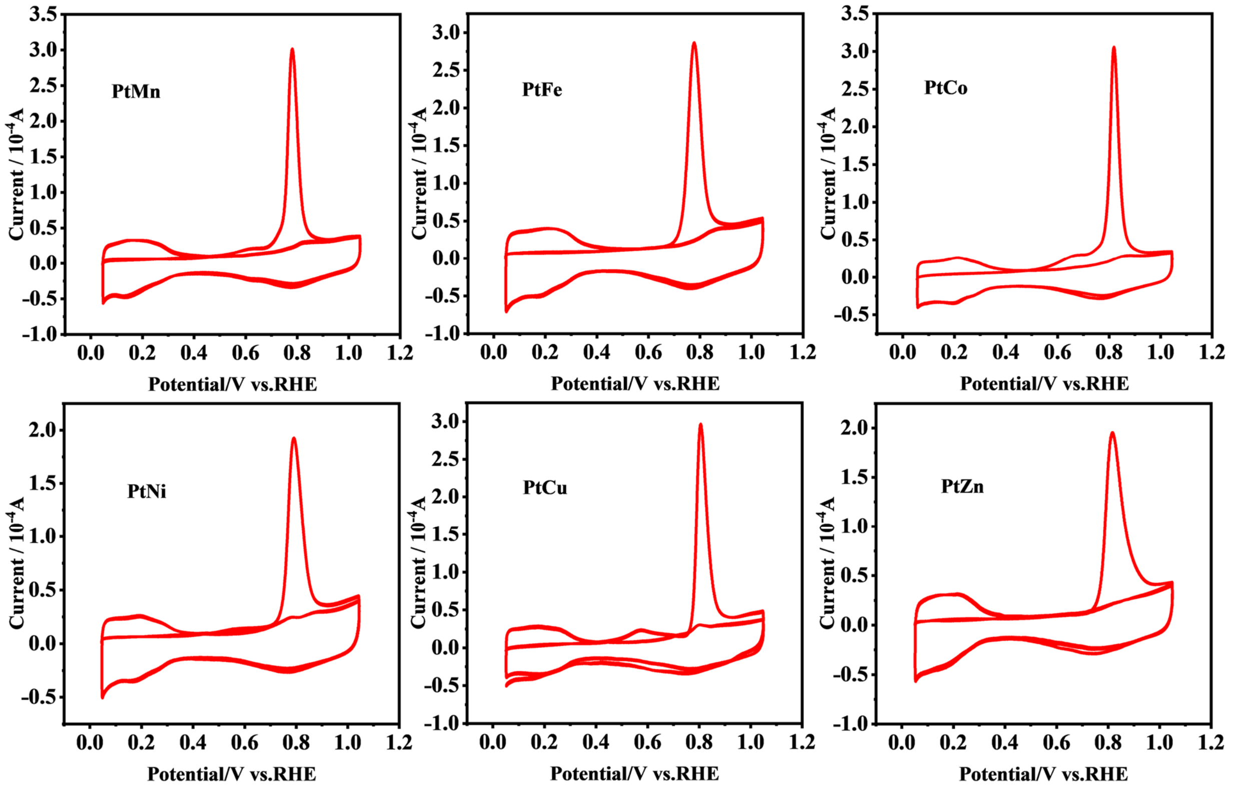

Supplementary Figure 23. CO-stripping curves of the IMCs catalysts for the ECSA measurements. The corresponding values of ECSA were listed in Supplementary Table 6.

Supplementary Table 1. Average particle sizes of the PtCo samples prepared with different molecule additives at 700 °C.

Sample	XRD size (nm)	STEM size (nm)
PtCo-Additive free	14.9	12.9
PtCo-BZF	12.4	9.5
PtCo-GLU	9.6	8.8
PtCo-SAc	8.3	7.7
PtCo-APR	6.4	5.7
PtCo-SGC	4.8	5.5
PtCo-DCDA	5.5	5.4
PtCo-MUA	4.3	3.4
PtCo-MBM	3.5	3.0
PtCo-MPA	2.4	2.3
PtCo-STG	2.8	2.5

Supplementary Table 2. Average particle sizes of the 18 Pt-IMCs catalysts prepared with and without STG additive from XRD results. The optimal annealing procedure and ordering degree of the Pt-IMCs prepared with STG additive were also listed.

Sample	XRD size (nm)		Optimal annealing procedure	Ordering degree (%)
	With STG	STG-free		
Pt_3Al	4.9	9.7	900/2h	27[a]
Pt_3Ti	4.3	8.8	900/2h-600/6h	35[a]
Pt_3V	5.4	10.4	1000/2h-600/6h	35[a]
Pt_3Cr	4.3	8.7	900/2h-600/6h	82[a]
Pt_3Mn	4.1	8.3	800/2h-600/6h	75[a]
PtMn	4.5	10.0	900/2h-600/6h	49[b]
Pt_3Fe	4.8	8.5	900/2h-600/6h	73[a]
PtFe	3.5	8.9	900/2h-700/6h	76[c]
Pt_3Co	5.2	8.2	900/2h-600/6h	82[a]
PtCo	3.3	9.2	900/2h-600/6h	72[c]
PtNi	3.4	8.6	900/2h-550/6h	53[c]
PtCu	3.0	8.7	800/2h-600/6h	—
$PtCu_3$	3.5	12.2	900/2h-600/6h	34[d]
PtZn	4.7	9.5	900/2h-600/6h	51[b]
Pt_3Ga	5.4	11.1	900/2h	77[a]
Pt_3Ge	5.6	11.3	900/2h	—
Pt_3In	4.2	11.5	900/2h	96[a]
Pt_3Sn	4.7	8.3	900/2h	94[a]

[a] $S_{(110)}/S_{(111)}$

[b] $S_{(110)}/[S_{(111)}+S_{(200)}]$

[c] $S_{(110)}/[S_{(111)}+S_{(200)}+S_{(002)}]$

[d] $I_{(110)}/I_{(111)}$

Supplementary Table 3. Type and dosage of Pt and non-Pt precursors used for the synthesis of Pt-IMCs libraries.

Sample	Pt precursor		Non-Pt precursor	
	Type	Dosage (mmol)	Type	Dosage (mmol)
Pt_3Al	$H_2PtCl_6·6H_2O$	0.13	$AlCl_3·6H_2O$	0.052
Pt_3Ti		0.203	$TiCl_4$	0.068
Pt_3V		0.202	VCl_3	0.067
Pt_3Cr		0.202	$CrCl_3·6H_2O$	0.067
Pt_3Mn		0.131	$MnCl_2·4H_2O$	0.052
PtMn		0.171	$MnCl_2·4H_2O$	0.206
Pt_3Fe		0.2	$FeCl_3$	0.067
PtFe		0.171	$FeCl_3$	0.205
Pt_3Co		0.2	$CoCl_2·6H_2O$	0.067
PtCo		0.168	$CoCl_2·6H_2O$	0.252
PtNi		0.169	$NiCl_2·6H_2O$	0.203
PtCu		0.166	$Cu(NO_3)_2·3H_2O$	0.182
$PtCu_3$		0.111	$Cu(NO_3)_2·3H_2O$	0.4
PtZn		0.165	$Zn(NO_3)_2·6H_2O$	0.197
Pt_3Ga		0.132	$GaCl_3$	0.053
Pt_3Ge		0.132	$GeCl_4$	0.053
Pt_3In		0.135	$InCl_3$	0.054
Pt_3Sn		0.135	$SnCl_2·2H_2O$	0.054

Supplementary Table 4. The atomic ratio results obtained by EDS mapping of Supplementary Figures 4-8.

Sample	Atomic ratio (%)		
	Pt	M	S
PtFe	47.4	42.5	10.1
PtCo	47.5	45.2	7.3
PtNi	47.2	44.1	8.7
Pt_3Co	71.8	23.7	4.5
PtCu	54.8	45.2	--

Supplementary Table 5. Average particle sizes from XRD and the ordering degree of additional PtCo IMCs catalysts prepared with STG additives, including the ones prepared at different temperatures, the one synthesized in grams scale, and the one with a high metal loading of 45 wt%.

Sample	XRD size (nm)	Ordering degree (%)
PtCo-750ºC	2.3	53
PtCo-800ºC	2.5	63
PtCo-900ºC	3.6	72
PtCo-1000ºC	4.5	79
Large-scale PtCo	4.3	65
45 wt% PtCo	4.7	56

Supplementary Table 6. ECSA, mass activity, and specific activity of the commercial TKK-30 wt% Pt/C and the STG-assisted PtM IMCs catalysts. The mass activity and specific activity were obtained at 0.9 V vs RHE, and the electrochemical tests were measured in thin film-RDE (10 μg catalyst on 0.196 cm^2 disk).

Sample	ECSA (m^2 g_{Pt}^{-1})	Mass activity ($A\ mg_{Pt}^{-1}$)	Specific activity ($mA\ cm^{-2}$)
Pt/C	72.5	0.35	0.48
PtMn	75.1	1.12	1.49
PtFe	86.7	2.18	2.51
PtCo	70.1	2.25	3.21
PtNi	63.4	2.11	3.33
PtCu	74.8	1.18	1.57
PtZn	78.8	0.88	1.12

Supplementary Table 7. Calculated surface strain of the five $L1_0$ PtM IMCs catalysts. Negative values indicate compression strain.

Sample	Surface strain (%)
PtMn	-1.83%
PtFe	-3.84%
PtCo	-8.24%
PtNi	-8.97%
PtZn	-2.74%

Supplementary Table 8. A detailed comparison of MEA performance of STG-assisted PtCo, Umic-30 wt% PtCo, TKK-30 wt% Pt/C, and TKK-30 wt% Pt/C-700 in H_2-air single-cell test.

**Sample**	**Current density ($mA\ cm^{-2}$) 80 °C/100%RH/150 kPa_{abs}**		**Power density ($W\ cm^{-2}$) 80 °C/100%RH/150 kPa_{abs}**		**Mass activity at 0.9 V ($A\ mg_{Pt}^{-1}$)**			**Voltage loss at 0.8 A cm^{-2} (mV)**		**Rated power density ($W\ cm^{-2}$) 94 °C/65%RH/250 kPa_{abs}**	
	at 0.8 V	at 0.6 V	at 0.8 V	at 0.6 V	initial	After ADT	DOE 2025 target			initial	ADT
TKK-30 wt% Pt/C	250	1470	0.20	0.88	0.32	0.15	≥ 0.44 A mg_{Pt}^{-1}<br>≤ 40% loss after ADT	83	DOE 2025 target: ≤ 30 mV loss after ADT	0.79	0.49
TKK-30 wt% Pt/C -700	216	1390	0.17	0.83	0.28	0.19		81		0.73	0.51
Umic-30 wt% PtCo	395	1659	0.32	0.99	0.86	0.58		76		0.94	0.71
STG-assisted PtCo	412	1821	0.33	1.09	1.08	0.81		21		1.17	1.03
